# Algal-based membrane bioreactors: a sustainable Frontier for removing emerging pollutants from wastewater

**DOI:** 10.1039/d5ra08065g

**Published:** 2025-12-10

**Authors:** Nadeem Raza, Zeeshan Ali, Suryyia Manzoor, Abdelmonaim Azzouz, Khalid Aziz, Sarfaraz Hashim, Mohamed Khairy, Mohamed E. Salem, Anis Ahmad Chaudhary

**Affiliations:** a Department of Chemistry, College of Science, Imam Mohammad Ibn Saud Islamic University (IMSIU) Riyadh Kingdom of Saudi Arabia; b Department of Climate Change, MNS-University of Agriculture Multan Pakistan zeeshan.ali@mnsuam.edu.pk; c Institute of Chemical Sciences, Bahauddin Zakariya University Multan Pakistan; d Laboratory of Water, Research, and Environmental Analysis, Faculty of Sciences, Abdelmalek Essaadi University Tetouan Morocco; e Department of Biology, College of Science, Imam Mohammad Ibn Saud Islamic University (IMSIU) Riyadh Kingdom of Saudi Arabia; f Department of Agricultural Engineering MNS University of Agriculture Multan Pakistan

## Abstract

Algal-based membrane bioreactors (AMBRs) have gained attention due to the increasing need for sustainable wastewater treatment methods. These reactors use membrane filtration and algal–bacterial activities to remove pollutants and recover biomass at the same time. This review provides a critical overview of the latest progress in AMBR systems regarding their configuration, membrane materials, pollutant removal mechanisms, and operation performance. Special emphasis has been laid on the chemical and biochemical mechanisms of nutrient and emerging pollutants (EPs) removal, involving adsorption, biodegradation, and photo-oxidative transformation in the algal–bacterial consortia. Further discussion covers the roles of membrane chemistry, surface modification, and fouling behavior concerning physicochemical interactions between EPs, algal metabolites, and membrane surfaces. Comparison data relying on removal efficiencies among different types of AMBR will be analyzed for highlighting the effect of algal strain, reactor design, and operating parameters. Moreover, emerging anti-fouling strategies, economic considerations, and perspectives on biomass valorization is summarized. Contrasting to most of the earlier reviews, this contribution provides a chemistry-oriented synthesis that links material properties to bioprocess mechanisms and reactor performance and may guide future research and optimization of AMBR technology for sustainable wastewater management.

## Abstract 2

Algal-based membrane bioreactors (AMBRs) have gained attention due to the increasing need for sustainable wastewater treatment methods.

## Graphical abstract

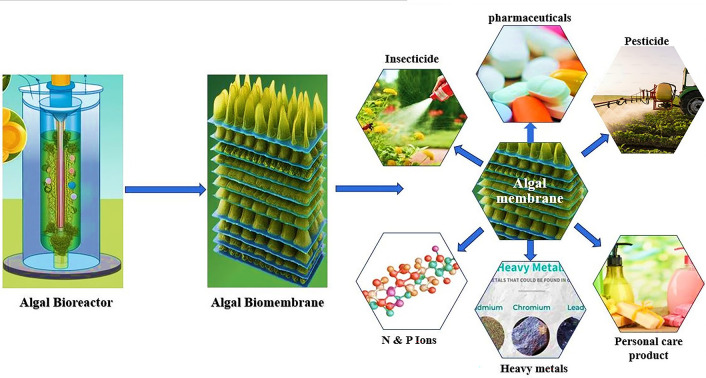

## 1. Introduction

The rapid and uncontrolled expansion of the global population has led to substantial industrial growth to meet the daily life demands. Consequently, large-scale industrialization not only generates millions of tons of hazardous pollutants, posing serious environmental threats but also requires vast amounts of clean water for power generation and the production of various everyday products.^1^ According to reports from the European Investment Bank, approximately 3.8 × 10^5^ billion liters of municipal wastewater are generated globally, with projections indicating an increase of 24% by 2030 and 51% by 2050.^2^ Notably, about 48% of the total wastewater produced globally is discharged into different environmental compartments without adequate treatment.^3^ Therefore, keeping in mind the presently available data of water resources worldwide, it is critical to adopt innovative methods and approaches for improving water cycle management in both public and industrial sectors.^4^ Furthermore, to fully recognize the value of water, sustainable strategies must be integrated throughout the entire water cycle. Additionally, wastewater recovery should be regarded as a valuable resource, and the deployment of advanced technologies is essential to facilitate its effective reuse.^5^

All the approaches deployed for the removal of pollutants such as pharmaceuticals, soap, oils, food, human waste, heavy metals, insecticides, and organic solvents contained in wastewater can be grouped into four main classes including: (a) physical (filtration, aeration, and sedimentation), chemical (advanced oxidation, adsorption, coagulation, ion exchange, and photocatalysis), mechanical (ceramic membrane technology and sand filter technology), and biological (aerobic, anaerobic, and composting).^6^ Among several wastewater treatment technologies, algal-based membrane bioreactors (AMBRs) represent an emerging and integrative option that merges biological and physical processes, thus offering improved effluent quality and resource recovery.^7^ In the last two decades, AMBRs have gained significant attention due to their ability to sustain high biomass concentration, achieve effective solids retention, and operate under relatively simple system configurations while yielding consistent effluent quality from municipal and industrial wastewaters.^8^ Resultantly, AMBRs are now recognized as an advanced wastewater treatment technology owing to their multiple advantages, including high decontamination efficiency, resistance to high organic loading, effective separation of inorganic and organic pollutants, low sludge production rate associated with extended sludge retention time (SRT) and minimized hydraulic retention time (HRT).^9,10^ A longer SRT facilitates the development of slowly growing bacteria benefiting the enhanced degradation of nitrogen-based species. Despite these advantages, AMBRs are not without limitations, particularly in terms of high operating and capital costs, membrane fouling, and significant energy demands.^11^ Therefore, for the successful commercial applications of AMBRs, it is essential to address these challenges to enhance their overall performance.

A typical AMBR system consists of two main components: (a) biological processes unit, where microorganisms degrade matter present in wastewater, and (b) membrane filtration unit, such as micro-filtration or ultra-filtration, which removes solids and microorganisms suspended in treated wastewater. Notably, biomass degradation occurs within the bioreactor tank, while the purification of treated water; removing microorganisms and suspended particles takes place in the membrane module. As a result, AMBR systems produce highly treated effluent that can either be safely discharged into the environment or reused for various applications.^12^ In this review, “algal-based membrane bioreactors” refer specifically to the systems that integrate membrane separation with algal or algal–bacterial processes for wastewater treatment. Conventional photobioreactors (PBRs) without membrane coupling are discussed only where their findings help explain algal metabolic behavior or pollutant removal mechanisms relevant to AMBR operation.

The objective of this review article is to evaluate the chemistry and performance of membrane materials, reactor and membrane-algae technological configurations, mechanistic pathways governing removal of emerging pollutants. Different components and working principles of AMBRs. The potential of AMBRs in the elimination of commonly occurring emerging pollutants (EPs) including pharmaceuticals, insecticides, personal care products, heavy metal ions, and nutrients in aqueous environments are discussed. Different components, and working principles of AMBRs are discussed. Various types of AMBRs, including photobioreactors (PBRs), microalgal-activated sludge membrane bioreactors (MAS-MBR), moving bed biofilm reactor membrane bioreactors (MBBR-MBR), and submerged membrane bioreactors (SMBRs) are also discussed. The performance of AMBRs is examined in relation to several key parameters, including light intensity, pH, temperature, algal biomass, mechanical aeration, HRT, SRT, inhibitory chemicals, algal–bacterial consortia, and reactor architecture. Finally, the potential limitations and future challenges of this technique are elucidated comprehensively.

## 2. Techniques utilized for wastewater treatment

Various approaches are used to remove pollutants, including pharmaceuticals, soap/detergents, oils, food waste, human waste, heavy metal ions, dyes, insecticides/pesticides, and organic solvents in wastewater. These methods can be categorized into four main classes: physical (filtration, aeration, and sedimentation), chemical (advanced oxidation, adsorption, coagulation, ion exchange, and photocatalysis), mechanical (ceramic membrane technology and sand filtration), and biological (aerobic, anaerobic, and composting processes).^13,14^ To focus the discussion on wastewater treatment using algal membrane reactors, phycoremediation and phytoremediation approaches will be discussed.

Phycoremediation involves algae including microalgae, macroalgae, and cyanobacteria, to remove pollutants and nutrients from wastewater and other aquatic environments.^15^ As phycoremediation lowers the overhead costs involved with nutrient delivery, it may be a more affordable method for removing emerging pollutants from wastewater and has gained popularity as the best method for eliminating emerging pollutants from wastewater in recent years.^16^

Algae, photosynthetic microorganisms that can be unicellular or multicellular, have gained tremendous focus for their role in sustainable wastewater treatment. Further, their capacity to eliminate nutrients (*i.e.*, phosphorus and nitrogen) *via* biological processes including assimilation and adsorption has made them valuable for wastewater treatment. Additionally, algae can eliminate organic and inorganic toxins *via* several mechanistic process such as bioaccumulation and biosorption.^17^ They have also been demonstrated to be highly effective in the elimination of heavy metal ions, emerging organic pollutants, and pathogens from wastewater.^18,19^ The presence of polysaccharides in algae, which can absorb micropollutants, makes them superior to bacteria and fungi for bioremediation.^20^ Algal polysaccharides, especially alginate and cellulose, enhance the attachment and disposal of numerous harmful substances, particularly heavy metals and organic contaminants, *via* biosorption methods that are affected by their distinct cell wall architectures.^21^

**Fig. 1:**
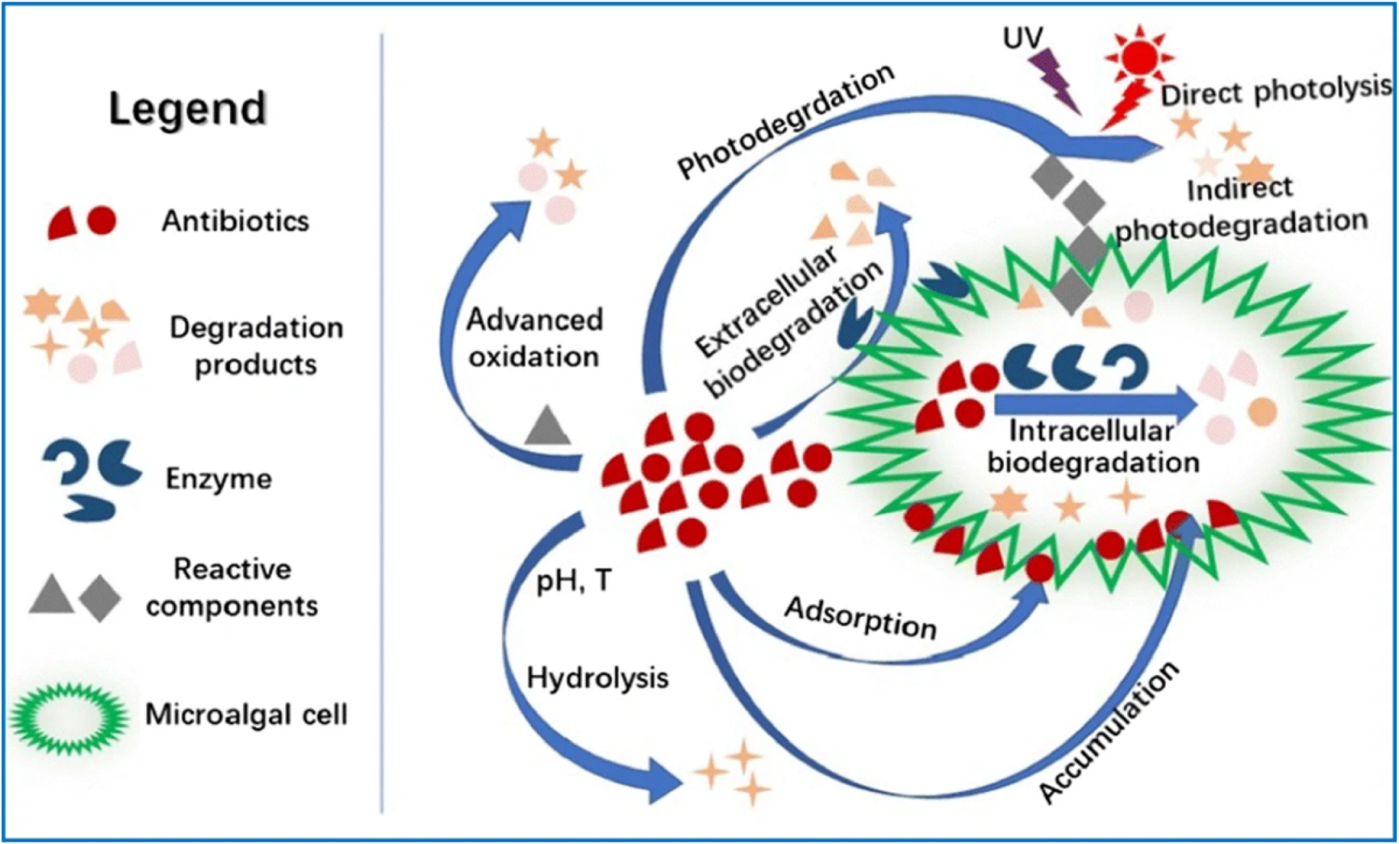
Schematic illustration of the mechanism of emerging pollutant removal by microalgae, reproduced from ref. 28 with permission from Elsevier, *Chemosphere*, vol. **238**, p. 124680, Copyright 2020.

Macroalgae used in phycoremediation are also effective in removing heavy metal ions and chemical dyes from different segments of aquatic system. However, unlike macroalgae, unicellular organisms such as microalgae exhibit significantly faster growth rates and greater resistance to harsh environmental conditions, including high temperatures, salinity, and nutrient stress.^22^ They also demonstrate strong resistance to EPs such as pharmaceutical drugs, organic solvents, dyes, pesticides, and heavy metal ions.^23^ Moreover, most microalgae can grow heterotrophically, mixotrophically, or autotrophically.^24^ Their unique genetic, enzymatic, and chemical variety, which differentiates them from plants, fungi, and mammals, further enhances their phycoremediation potential. The removal of EPs through phytoremediation involves multiple processes, including (a) biosorption, (b) bio-uptake, (c) bioaccumulation, (d) biodegradation, and (e) photo-deterioration as summarized in Fig. 1.^25–28^ These biological processes/approaches, used to remove EPs, are unique and effective. However, deployment of a specific approach requires a distinct biological agent or mechanism to reduce environmental pollution, which contributes to long-term cleanup solutions.^29^ For example, biosorption is the passive absorption of EPs by biological organisms like algae and fungi *via* processes such as ion transfer, adsorption, and complexity, resulting in the elimination of heavy metals and organic pollutants from water. Bio-uptake is the continual transport of EPs into living things, in which they can be processed or stored, hence lowering the amount of pollutants in the surroundings.^30^ In bioaccumulation algae absorb and accumulate contaminants from their surroundings gradually, resulting in larger intrinsic levels compared to those in the medium around them, which can endanger the food system chain. Further, biodegradation is more beneficial as it involves disintegration of EPs into simpler, harmless molecules, which frequently results in full mineralization to carbon dioxide and water, therefore recovering the integrity of the environment. In case of photo-deterioration, the decomposition of harmful substances is accomplished by photochemical processes promoted by sunshine, which results in the decomposition of more complicated organic molecules into simpler and less hazardous chemicals. Though these processes are successful in removing pollutants, there are still obstacles in optimizing their effectiveness and flexibility for commercial applications in environmental restoration.^31^

In addition to phycoremediation using algae for water detoxification, extensive research has explored the use of seaweeds for wastewater treatment through phytoremediation approaches.^32,33^ However, seaweeds have limited applications due to their specific culture requirements, such as salinity, low temperature, and pH tolerance, which pose challenges for researchers.^34^ Furthermore, their relatively slow growth rate and the need for abundant and sustainable biomass supplies further constrain their widespread use.^35^

Phytoremediation and phycoremediation are recognized as two environmentally benign procedures for disinfecting zones of contamination, however they use distinct biological substances. Phytoremediation uses larger plants to collect, settle, or disintegrate contaminants from soil and water through methods including phytoextraction, rhizofiltration, and phyto stabilization. Phytoremediation is very successful for a wide range of harmful substances, comprising heavy metals and organic substances, and it is financially feasible due to its capacity to harvest and use the biomass generated.^36^ In contrast, phycoremediation uses algae to absorb and collect heavy metals and micronutrients from waterways, effectively decreasing pollution levels. Algae's fast growth and production of biomass enable the development of alternative sources of energy, enabling phycoremediation a multipurpose technique. Conclusively, both approaches are potential options for long-term environmental restoration, but they employ different biological processes and thus differ in applications.

While phytoremediation and phycoremediation can provide major advantages, there are still hurdles to improve their efficiency and scalability. For example, the performance of these approaches can be enhanced by considering environmental circumstances and the types of contaminants present, encouraging further studies and improvement to enhance their practical applicability.^12^

## 3. Working mechanism of algal based membrane bioreactors

The growing need for plentiful supply of pure water worldwide and the shortcomings of conventional treatment techniques have made AMBRs an appealing option for the elimination of emerging contaminants (ECs) from water. Historically, the use of algae in treating wastewater has been identified for durability and effectiveness, especially for tackling contaminants such as pharmaceuticals and personal care products, which traditional approaches frequently struggle to eliminate.^37^ AMBRs take advantage of algae's distinctive characteristics, integrating biological decomposition and physical filtering to increase absorption of pollutants and biomass yield. With the passage of time, AMBRs technology has received significant advancements in terms of modifications in configuration and operational variables targeted at enhancing efficiency and reducing fouling.^38^

In a standard AMBR system, a membrane separation unit is integrated with biological treatment involving bacteria that need oxygen and dissolved organic carbon for growth. A membrane separates microbial biomass from the effluent while filtering out bacteria and suspended particles. Although conventional MBRs effectively remove organic carbon from wastewater, but they struggle to eliminate nitrogen and phosphorus.^16^ To address this limitation, a new generation of AMBR is being developed to enhance nutrient removal through effective reduction in total suspended solid, biological oxygen demand (BOD), and chemical oxygen demand (COD).^39,40^

The biological treatment process in AMBRs begins with the utilization of algae.^31^ As photosynthetic organisms, algae use light energy to absorb nutrients and organic substances from wastewater.^41^ Through photosynthesis, algae generate oxygen, which can help keep the environment aerobic and speed up the decomposition of organic materials.^42^ Additionally, phycoremediation of wastewater can be benefited with several key advantages in terms of enhanced removal efficiencies, minimal energy usage, and biomass generation essentially required for fertilizers and/or for biogas generation.^43^ The second phase in AMBRs operation is the usage of membranes for physical separation. These membranes retain biomass inside the system thus improving the removal efficiency of contaminants from water. Further, membranes are capable to stop the release of surplus biomass into the ecosystem which can lead to eutrophication and several other allied environmental issues.^44^ Furthermore, the usage of algae can improve the efficiency with which pollutants are removed, while the deployment of membranes can lower the environmental imprint of standard treatment techniques.^45^

In AMBRs, nutrient removal occurs through absorption and chemical precipitation of nitrogen and phosphorus by algae. Additionally, algae can produce persistent chemical phosphates by forced flocculation operations, in which algal cells aggregate into bigger flakes for smooth sedimentation. This method is mostly helped by the presence of flocculants, such as ferric chloride or calcium phosphate, that bind to algal cells and extrinsic organic matter, increasing their interaction and bonding, thereby encouraging floc development.^46^

Unlike conventional treatment methods, algal-based remediation does not require additional chemicals, as phosphorus can be recovered as a valued byproduct from algae biomass. Consequently, algae-induced phosphorus precipitation is considered an eco-friendly technique suitable for phosphorus recovery from aqueous environments. Beyond wastewater detoxification, AMBRs also provide an alternative source of biomass for biofuels, fertilizers, and other valuable applications.^47^

It is also worth noting that phycoremediation is not a new concept, as it is naturally occurring in ecosystems such as lakes and wetlands for decades, helping to maintain ecological balance. However, the integration of algae into AMBRs for wastewater detoxification is a relatively recent advancement.^44^

### 3.1. Components of an algal based membrane bioreactor

**Fig. 2:**
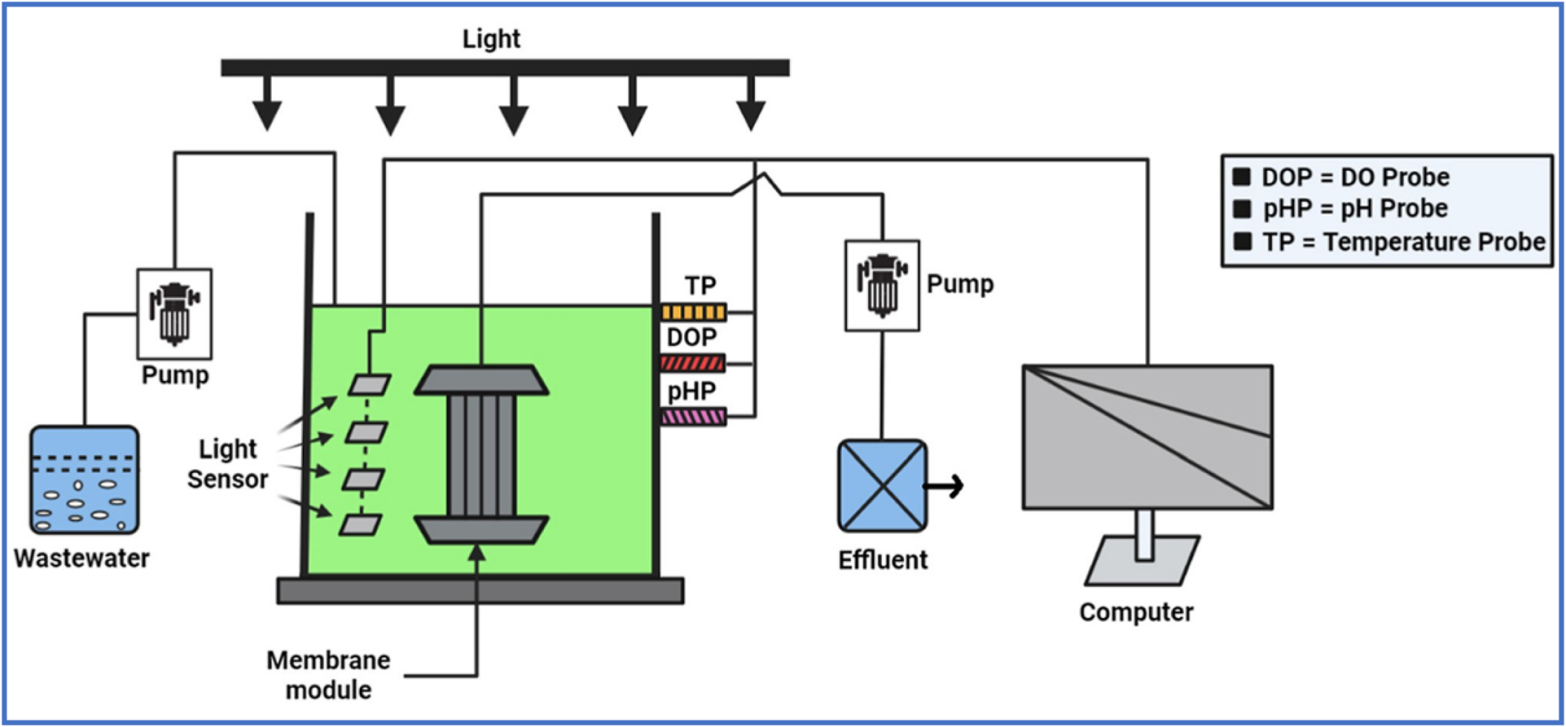
A systematic diagram of algal based membrane bioreactors, reproduced from ref. 44, with permission from Elsevier, *Chemosphere*, vol. **336**, p. 139291, Copyright 2023.

The components of an AMBR may vary depending on its specific design and functional requirements. However, a typical AMBR consists of the following major components, as depicted in Fig. 2.

(1) Light source: this could be either natural sunlight or artificial lighting, such as LED lights.^48^ In algae bioreactors, light is essential for photosynthesis and optimal algae growth.

(2) Culture vessel: this is the container where algae grow, which can be made of various materials, including metal, glass, or plastic. Culture vessels come in different shapes, such as tubes, tanks, or bags.^49^

(3) Mixing and aeration system: a well-designed system for mixing and aerating the algae culture is crucial to avoid stratification and to provide oxygen for algae growth.^50^

(4) Nutrient delivery system: this mechanism supplies essential nutrients, such as fertilizer or wastewater, to support algae growth.^51^

(5) Filtration system: a centrifuge or filtration system is used to separate and collect algae from the culture.^52^ A crucial component of AMBRs is the membrane, which acts as a physical barrier to stop bacteria and algal biomass from entering the water, thereby ensuring high-quality effluent. The assortment of a well-suited membrane is essential for impactful performance of AMBRs. An effective membrane should be resistive towards challenging wastewater treatment environments, such as fouling, scaling, and chemical attack. To this end, membranes exhibiting small pore size are more appropriate, as they can efficiently retain algae biomass and bacteria while permitting the clean water to pass.^53^

**Fig. 3:**
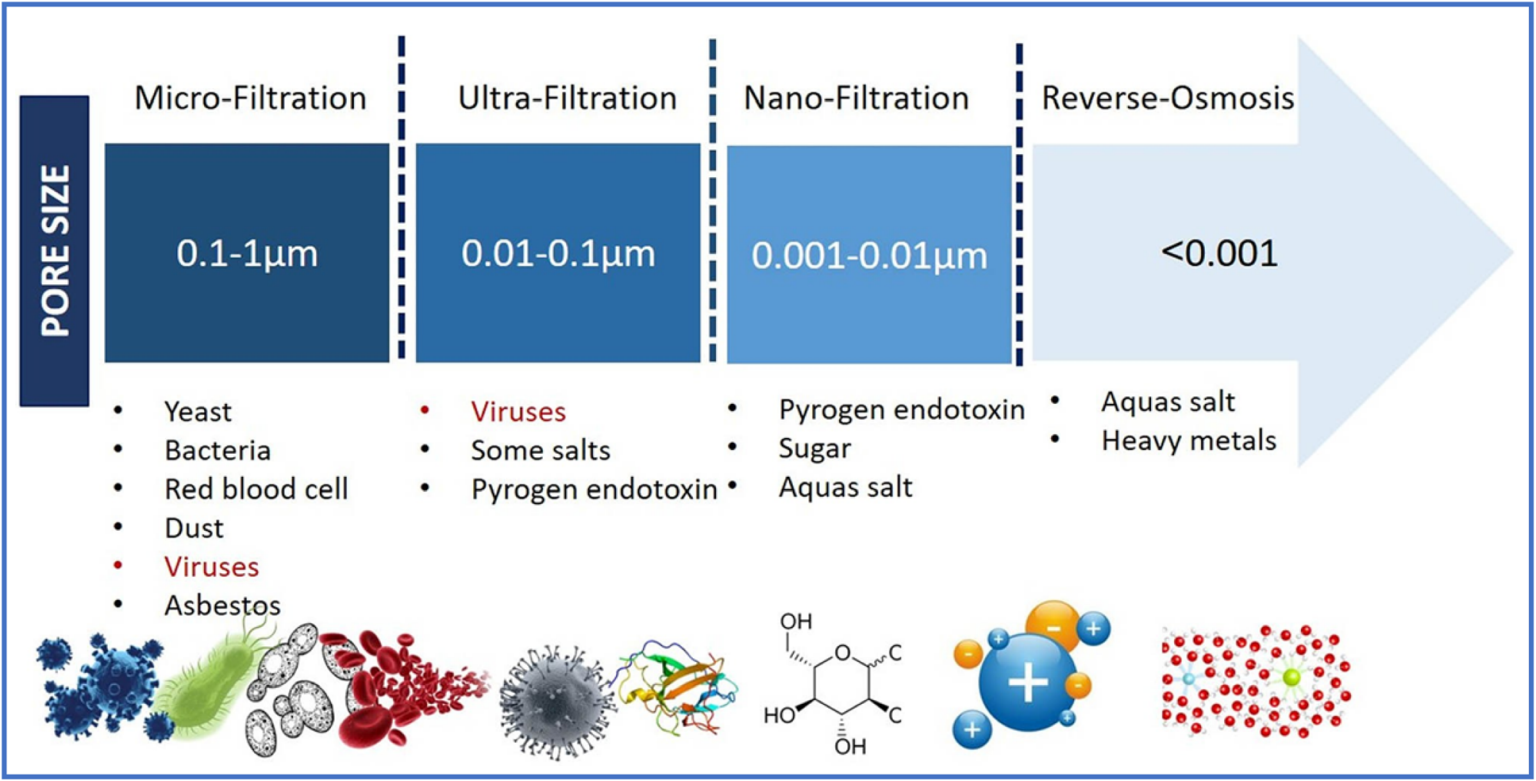
Classification of membrane processes based on pore size and removal criteria, reproduced under a Creative Commons CC BY Attribution 4.0 International License.^57^

Nowadays, membranes of various composition, such as polymeric, ceramic, and composite membranes, are commonly utilized in AMBRs. Among these, polymeric membranes are extensively deployed owing to their cost-effectiveness, high elasticity, and comfort in regeneration.^54^ In contrast, ceramic membranes are resistant to chemical deterioration and have strong mechanical strength and lifespan and they demonstrate exceptional stability.^55^ Likewise, composite membranes, which are composed of diverse materials, offer a number of advantages over single-component membranes, including improved permeability and resistance to fouling. However, their extensive exploitation is limited by the complexity of their manufacturing process and the high costs associated with it.^56^ It is well established that the pore size of the of AMBRs determines the nature of filtration from micro to nanofiltration and controls the quality of treated water (Fig. 3).

Monitoring and control system: this system includes sensors and controllers that track and regulate key parameters such as pH, light intensity, temperature, and other factors crucial for algal growth.^58^

(6) Power and control systems: these comprise electrical and electronic components that supply power to bioreactor and its control systems.^59^

### 3.2. Types of AMBRs

#### 3.2.1. Selection of algal biomass

##### 3.2.1.1. Algal–bacterial symbiosis based AMBRs

In such bioreactors, microalgae and bacteria cohabit and interact in a confined and regulated environment. Through photosynthesis, sunlight and CO_2_ are absorbed by microalgae which generate organic matter and oxygen essentially required for bacterial growth.^60^ In return, bacteria facilitate the breakdown of organic matter and aid in the removal of nutrients and other pollutants from water.^61^ Notably, the membrane filtering process filters bacteria and suspended materials from being released, resulting in high-quality effluent. AMBRs have demonstrated significant promise for pollutants removal from wastewater along with biomass and energy as byproducts.^21^ In natural ecosystems, bacteria commonly co-assist algae in wastewater detoxication activities.^62^ However, investigating algal–bacterial interactions is challenging as algae and bacteria are inherently bonded to one another. Relative to an algal–bacterial system, solo algae systems have a relatively poor removal efficiencies in wastewater treatment. AMBRs may take full credit for the relationship between bacteria and algae and advantages of algal–bacterial symbiosis and membrane filtration.^63^

Because of their excellent nutrient removal efficiency, algae-activated sludge systems particularly those incorporating *Chlorella* strains—have received a lot of interest in recent years.^64^ Studies have reported that nutrient removal efficiency, exceeding 90% for ammonium and COD removal, is attributed to the symbiotic relationship between algae and bacteria cells.^65,66^ Furthermore, compared to activated sludge alone, algae–bacteria biomass demonstrated superior nitrogen absorption capabilities, whereas biomass containing only bacteria has demonstrated lower removal efficiencies relative to algae–bacteria biomass.^67,68^

##### 3.2.1.2. Mixed algae AMBRs

Mixed algae strains can be employed to improve wastewater treatment and biofuel generation.^60,69^ For instance, Radmehr *et al.* evaluated the impact of monospecific and mixed-algae culture on the efficiency of algae-sludge-MBRs using two microalgae strains (*Chlamydomonas* and *Selenastrum*) and their combination inserted into conventional-membrane-bioreactors (CMBRs).^70^ The mixed-algae membrane bioreactor performed best in terms of nutrient removal, chlorophyll-*a* content, and membrane fouling. The findings suggested that the bacterial communities contained in algae-MBRs and CMBR were altered, indicating that inoculation of algal strains would selectively favor members of bacterial strains that collaborated with algae strains. These interactions might be caused by bacteria and algae assisting each other's photoautotrophic and heterotrophic metabolisms *via* exchanges of oxygen, carbon dioxide, and other chemicals or vitamins (*e.g.*, thiamine, B12, biotin, *etc.*).

It is well acknowledged that the structure of the biomass microbial communities and diversified populations are closely connected to the performance of MBRs. Mixing specific algae strains in a single MBR not only reduces the possible toxic effects of high single-algae enrichment on bacterial community but also yields greater variety of microorganisms than single-algae inoculation. Algal mixed culture has also been used in membrane photobioreactors to attain sufficient treatment efficiency for N and P, as well as biomass productivity.^71^

#### 3.2.2. Design and mechanism of algal membrane bioreactors

##### 3.2.2.1. Photobioreactors

**Fig. 4:**
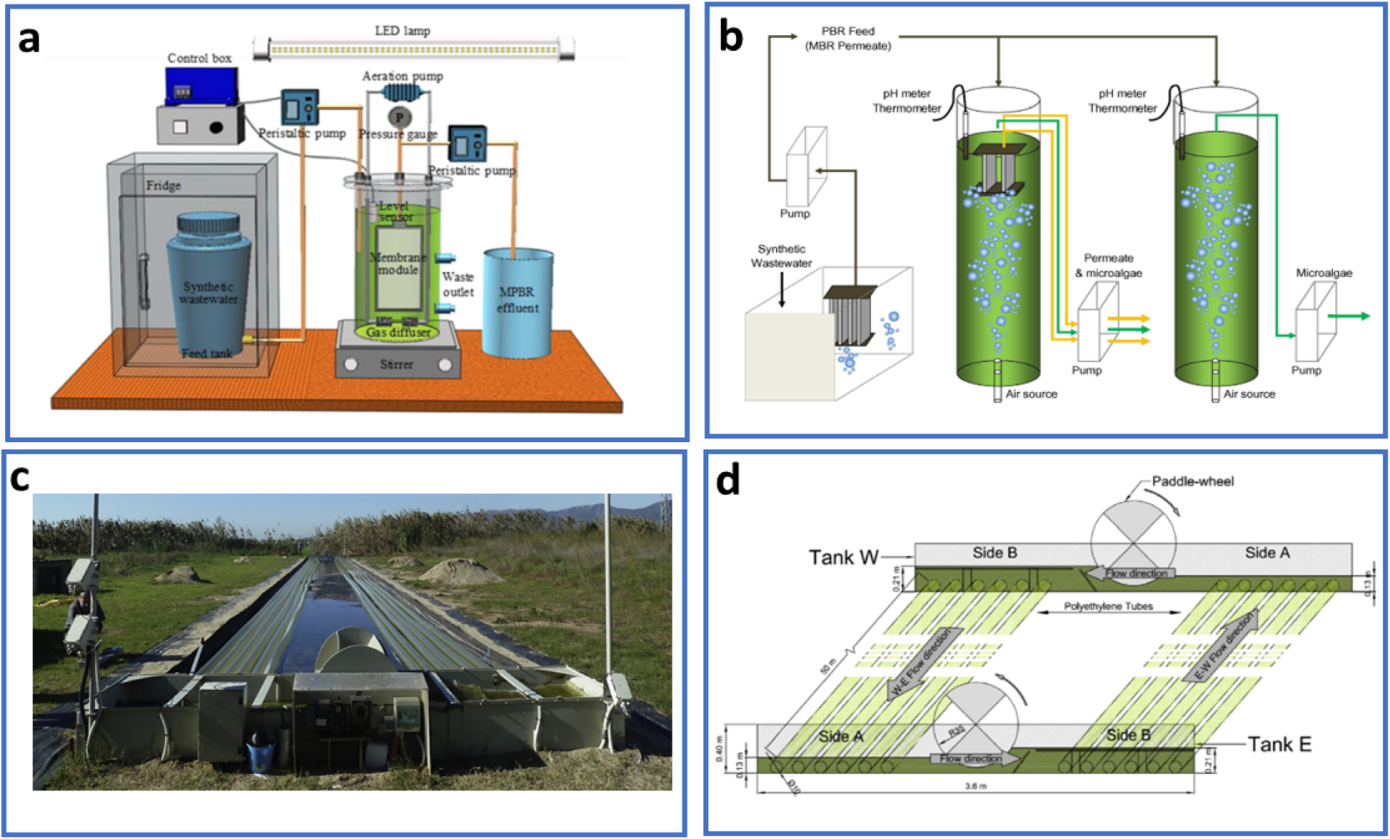
(a) Experimental setup of a typical MPBR reproduced under a Creative Commons CC BY Attribution 4.0 International License,^79^ (b) schematic illustration of bubble column equipped with an air bubble source at the bottom of PBR reproduced from ref. 81 with permission from Elsevier, *Bioresource Technology*, vol. **163**, p. 228, Copyright 2014, (c) full-scale hybrid tubular horizontal photobioreactor (HTH-PBR) at full capacity, and (d) flow sheet and sketch of different parts of the full-scale HTH-PBR reproduced from ref. 80 with permission from Elsevier, *Biosystems Engineering*, vol. **166**, p. 138, Copyright 2018.

Photobioreactors are closed systems where phototrophs are cultured while preventing direct material interaction between the cells and the environment.^72^ They effectively address a number of issues associated with open pond system, including heavy metal accumulation, microbial and insects pollution, and air pollution.^73^ Compared to open pond systems, photobioreactors are more compact and more space-efficient.^74^ They also offer precisely controlled conditions for the growth of microalgae and lower the risk of contamination by keeping an axenic algal culture.^75^ Higher biomass output per unit substrate and improved metabolic efficiency can be achieved in the regulated growing environment. However, photobioreactors face a number of design challenges due to their extremely limited practical applications and lack of economic viability from an industrial standpoint.^76^ Photobioreactor systems involve high initial and operational costs and specifically designed for fermentation under either solar light or artificial light, although artificial lighting demands substantial electricity, making it expensive.^77^ Consequently, outdoor photobioreactors are often preferred owing to their capacity to absorb solar radiations. Photobioreactors can be built out of glass or transparent plastic, and their light receiving structures are usually made up of a variety of tubes or flat panels, often known as solar receivers.^78^ Sustaining sufficient penetration of sunlight is crucial for the effective and continued fermentation process. Generally, photobioreactors run continuously at a temperature between 25 and 40 °C. Different geometries and configurations of photobioreactors (Fig. 4a–d) have been employed for diverse applications.^79–81^ These geometries may include: (a) continuous stirred tank bioreactors, (b) airlift bioreactors, (c) packed bed bioreactors, (d) fluidized bed bioreactors, (e) bubble column bioreactors, (f) tubular with different designs (horizontal, fence, and helical), and (g) vertical flat panel.

**Fig. 5:**
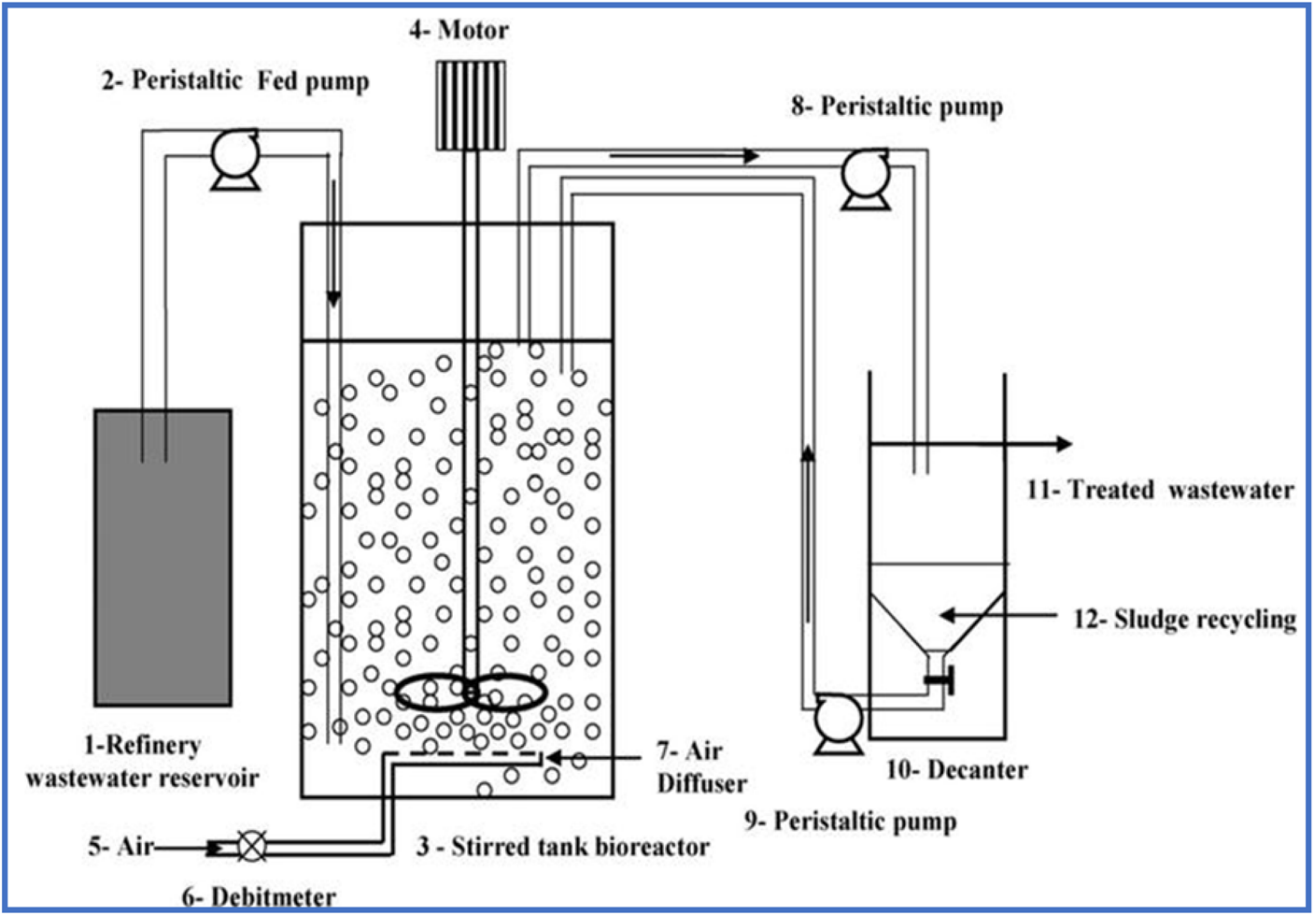
Schematic and working principle of a typical aerobic continuously stirred tank bioreactor reproduced under a Creative Commons CC BY Attribution 3.0 International License.^83^

Among these configurations, large-scale tubular photobioreactors (Fig. 4(c and d)) have been extensively employed in Germany and Israel for large scale production of *Haematococcus* and *Chlorella* species.^82^ Stirred tank photobioreactors (STPs) are more common owing to their simple design and are highly appropriate for shear sensitive microalgae cultivation as shown in Fig. 5.^83^ These systems are comprised of a glass tank continuously stirred by impellers or baffles, with CO_2_-enriched air bubbled into the system to deliver a carbon source for algae growth.^84,85^ Despite their simpler designs, STPs have certain drawbacks, including a low surface area/volume ratio limiting their light-harvesting capabilities.^86^ Efforts to improve STPs by incorporating microalgal–bacterial consortia have been reported. For example, an STP containing such a consortium achieved 95% removal efficiency of *p*-aminophenol with a HRT of 4 days.^87^ In another case, the use of STPs containing *Chaetoceros muelleri* resulted in relatively low removal efficiencies (33.1–36.5%) for pharmaceuticals such as carbamazepine, sulfamethazine, and tramadol.^84^

Photobioreactors have also been extensively employed for the removal of EPs in water treatment processes.^88,89^ For instance, nitrogen and phosphorous ions were eliminated from synthetic wastewater at original concentrations of 50 and 10 mg L^−1^, respectively, using a photobioreactor operated under optimized experimental conditions of 25 °C and 8.8 pH.^90^ A co-culture system containing the photosynthetic microalgae *Chlorella vulgaris* and the aerobic heterotrophic bacterium *Pseudomonas putida* achieved 80% removal efficiency of aforementioned ions in synthetic waste water system. In another study, a photobioreactor achieved approximately 70% removal efficiency of pharmaceutical pollutants from synthetic waste water with initial concentration of 0.332 mg L^−1^ at 8.1 pH.^84^

##### 3.2.2.2. Microalgal-activated sludge membrane bioreactor MAS-MBR

**Fig. 6:**
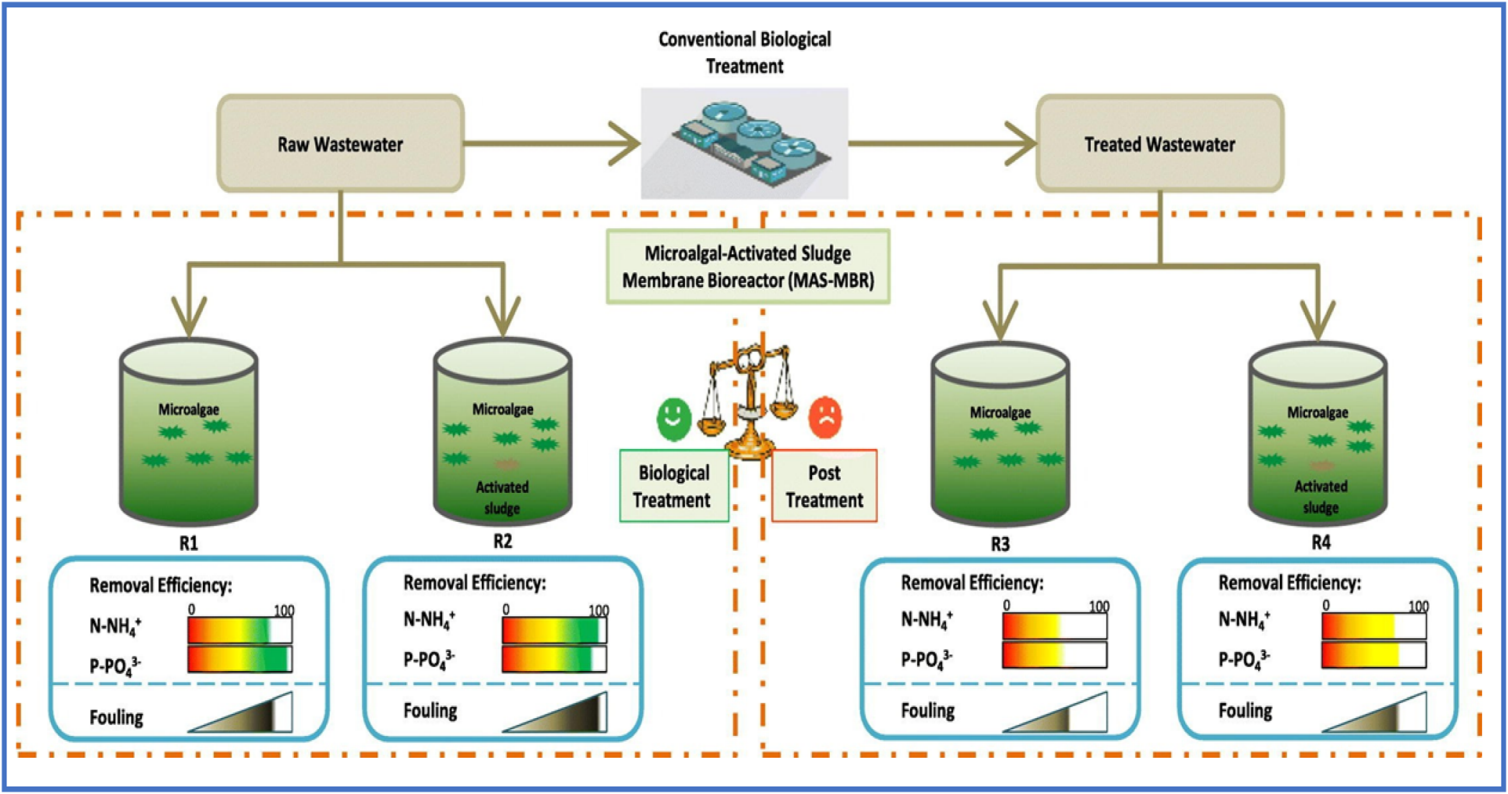
Working diagram of microalgal-activated membrane bioreactor reproduced from ref. 93 with permission from Elsevier, *Journal of water process engineering*, vol. **49**, p. 103069, Copyright 2022.

The microalgal-activated sludge membrane bioreactor (MAS-MBR) is a wastewater treatment system that combines microalgae and activated sludge within a membrane bioreactor, representing a promising new approach for municipal wastewater purification.^91^ MAS-MBR uses a membrane to filter out impurities, producing clean and reusable water for various applications such as domestic to industrial. A detailed representation of MAS-MBR is given in Fig. 6. Conventionally operated activated sludge biological treatment units have shown limited effectiveness (a significant concern since their implementation) in removing various ions, metals, and pesticides.^92^ Therefore, there is an urgent need to investigate the performance of MAS-MBR, either as a post-treatment process following traditional biological treatment or as a standalone biological treatment.

In one such study, a cylindrical continuous MAS-MBR system was tested through two different proportions of algae/sludge; (1) only microalgae and (b) 5 : 1 to investigate the removal efficiencies of EPs from raw and processed water.^93^ Cultivation of a mixture of *Chlorella vulgaris* and activated sludge in untreated wastewater over a 21 days operational period yielded the best results achieving ammonium and phosphorus elimination effectiveness reaching to 94.36 ± 3.5% and 88.37 ± 3%, respectively. Although the MAS-MBR has emerged as a prospective member for self-biological treatments, however, the membrane fouling remains a crucial challenge. High levels of membrane fouling are typically associated with the increased creation of the protein fraction of extracellular polymeric materials and carbohydrate fraction of soluble microbial compounds which can severely impact the system's long-term performance and operational stability.^94^

##### 3.2.2.3. Moving bed biofilm reactor membrane bioreactor (MBBR-MBR)

The development of MBRs and MBBRs in the second half of the 20th century has significantly advanced wastewater treatment research.^95^ Over the past ten years, research into MBR and MBBR technologies has gained considerable momentum, leading to numerous improvements and refinements over their original prototypes. Since MBBR procedures have a high potential for recovering and removing nutrients, they have shown great promise in the context of the circular economy.^96^ A typical MBBR biological treatment procedure, which allows both aerobic and anoxic processes, is comprised of a suspended solid and biofilm attached to plastic carrier that serve as a substratum for biofilm development. Additionally, in a continuously agitating MBBR, the deployment of high number of biofilm carriers enables the growth of bacterial biomass.^97^

**Fig. 7:**
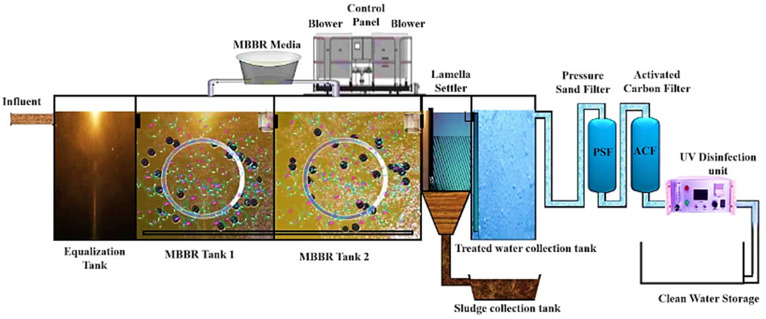
The working illustration of MBBR-MBR adopted under a Creative Commons CC BY Attribution 4.0 International License.^101^

Additionally, a number of studies have used the MBBR approach to achieve the efficient removal of newly EPs.^97–99^ However, MBBR alone might not be sufficient to meet the strict discharge limits in some situations requiring the treatment of high-strength wastewater.^100^ Thence, integration MBBR with MBR technology offers excellent potential for producing high-quality treated water.^95^ A schematic of the MBBR-MBR system is provided in Fig. 7. In an attempt to investigate heavy metals elimination, a MBBR-MBR system was utilized that effectively removed heavy metals such as zinc, lead, chromium, and iron, with removal rates of 96%, 92%, 85%, and 88%, respectively.^97^

##### 3.2.2.4. Submerged membrane bioreactor

Submerged membrane bioreactor (SMBR) techniques have been widely used for biological remediation of wastewater since its commercialization around 1990, typically employing microfiltration or ultrafiltration membranes.^102^ A typical SMBR operates a filtration process where polymeric microfiltration membranes, having pore size range of 0.1 to 0.4 µm, are submerged directly in the biomass, either in a separate tank or within the bioreactor itself.^103^ Vacuum pressure is used to facilitate the filtering process by applying suction to the interior of the membranes.

Periodic backwashing or the movement of large air bubbles along the membrane surface helps prevent membrane clogging. Moreover, the air across the membrane surface generates turbulence resulting in cleaning or scrubbing of the membrane which enables SMBRs to eliminate higher than 95% of COD. It is also of worth mentioning that the decrease in BOD values is significantly high in SMBR water treatment processes.^104^

**Fig. 8:**
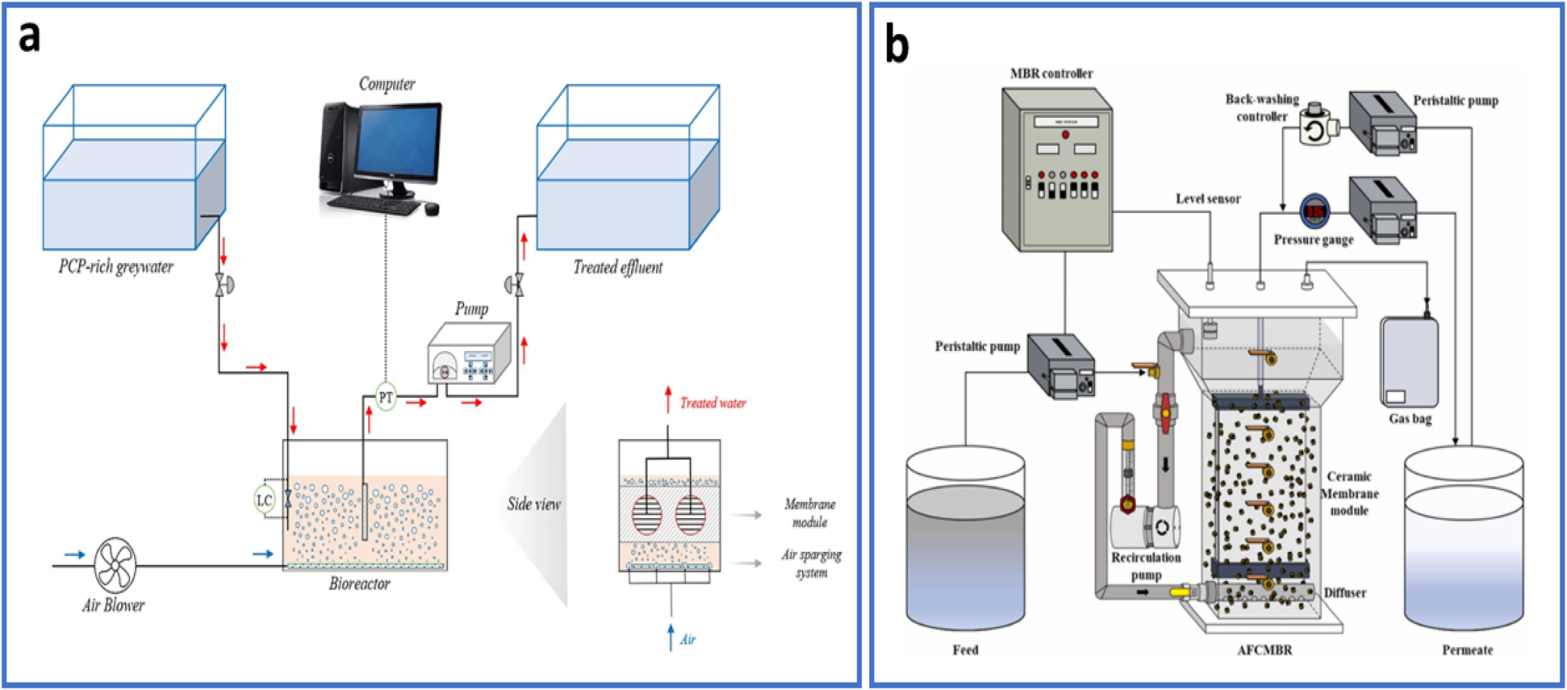
(a) The schematic of a typical SMBR system for wastewater treatment reproduced from ref. 105 with permission from Elsevier, *Journal of Environmental Chemical Engineering*, vol. **8**, p. 104432, Copyright 2020 and (b) schematic of AFCMBR reproduced from ref. 108 with permission from Elsevier, *Journal of Environmental Chemical Engineering*, vol. **11**, p. 109153, Copyright 2023.

SMBRs have been extensively employed for the removal of EPs in both synthetic and real wastewater samples. For example, one study utilized an SMBR for the removal of three personal care products (PCPs), including triclosan, methyl paraben, and propylparaben from synthetic wastewater.^105^ The relatively high removal efficiencies for the aforementioned PCPs were achieved; 98.20, 99.96 and 99.97%, respectively. The performance of AMBRs is closely tied to the chemistry and structure of the membranes used. The utilization of polymeric membranes relying on polyvinylidene fluoride, polyethersulfone/silica composite, and polyacrylonitrile dominate because of their hydrophilicity and mechanical flexibility, whereas ceramic and hybrid membranes offer higher thermal and chemical stability with lower fouling potential. Importantly, algae membrane interactions, influenced by surface charge, roughness, and hydrophobicity, control the formation and reversibility of the fouling layer.^106^ Consequently, advances in surface modification such as hydrophilic coatings, photocatalytic layers, and bio-inspired polymers are being developed to enhance flux recovery and selectivity. Integrating these material improvements within reactor design underscores the dual focus of AMBR technology: optimizing both biological activity and membrane-based liquid separation. In another study, a submerged ceramic flat membrane bioreactor was employed to treat coal chemical wastewater.^107^ This ceramic flat membrane bioreactor successful reduced ammonia nitrogen, COD, total phenol, and turbidity levels to below 3.03, 31.4, 3.76 mg L^−1^, and 0.4 NTU, respectively. Optimal pollutant removal was achieved at a HRT of 21 h, dissolved oxygen concentration of 3.2–4.0 mg L^−1^, and pH between 7.1 and 7.5. A working diagram of a standard SMBR is given in Fig. 8a. A slight modification to this system was proposed in the form of anaerobic fluidized bed ceramic membrane bioreactor (AFCMBR), illustrated in Fig. 8b.^108^ This system was employed to explore the relationship between HRT and methylparaben removal efficiency.

A standard anaerobic MBR (AnMBR) is constructed by combining an anaerobic bioreactor and membrane filtration unit to retain anaerobic microorganisms with reduced growth rate and generating high effluent (permeate) quality.^109^ However, an anaerobic fluidized bed membrane bioreactor (AFMBR) is made up of anaerobic fluidized bed bioreactor and submerged membrane filtration assembly normally comprised of granular activated carbon (GAC).^110^ Undoubtedly, GAC fluidization is energy intensive however, GAC particulates are easily detached and form small rubble which is among the potential foulants on the membrane surface of bioreactor and thus reducing overall efficiency of bioreactor.^111^ To address this issue, a flat-tubular ceramic membrane system was investigated which displayed significant potential for the efficient elimination of methylparaben.^108^

## 4. Factors affecting the performance of AMBRs

As discussed earlier that performance of a typical AMBR system in terms of wastewater treatment mainly depends on biological unit, consisting of algae and/or bacteria,^21^ which in turn is strictly affected by several factors, including temperature, pH, light intensity, algal biomass, mechanical aeration, hydraulic retention time, solid retention time, membrane fouling, inhibitory compounds, algal bacterial consortia, nutrient availability, and reactor design. Some algae species, for example, may respond to a specific pollutant more effectively, such as organic compounds, or heavy metals than others. Consequently, the choice of the right algal strain is critical for efficient elimination of certain pollutants from wastewater.^112,113^ The wide variation in removal efficiencies reported for similar pollutants arises primarily from differences in algal strains and associated bacterial communities, hydraulic retention times, light intensity, nutrient ratios, and membrane material properties. For example, *Chlorella vulgaris* often shows higher nutrient uptake under moderate illumination, whereas *Scenedesmus obliquus* performs better at elevated nitrogen levels. Similarly, the type of membrane (polymeric *vs.* ceramic) and its surface charge also influence pollutant adsorption and biofilm development. These factors collectively determine overall removal efficiency, as summarized schematically in Fig. 10.

### 4.1. Light intensity

Light is necessary for algae proper growth, and its intensity has a substantial impact on AMBR performance. To ensure accelerated algal biomass growth and pollutant elimination, optimal light intensity is required. Excessive light intensity, on the other hand, might cause photoinhibition, which can severely impact algal growth and activity, eventually reducing AMBRs performance. This photoinhibition is induced by a mechanism in which algae's photosynthesis ability decreases due to disruption of photosystem reaction site protein (D1) where light intensity approaches metabolic needs. The resulting harm can impede algal development and action, eventually reducing the functioning of AMBRs owing to lower biomass output and inefficient photosynthesis.^114^

### 4.2. pH and temperature

pH affects the degree of protonation and deprotonation, which in turn affects the surface charge of algal foulants and electrostatic attractive or repulsive forces with the membrane.^115^ Generally, low pH values create positive charges on the surface by enhancing the number of protonated amine groups, while high pH values deprotonate these groups, resulting in an overall negative surface charge. Relative to neutral and basic situations, repulsive interactions between transparent exopolymer particles (TEPs) and ultrafiltration membranes were significantly reduced by 41.7% under acidic conditions, supporting the attachment of free TEPs onto the membrane surface and thereby escalating the permanent membrane fouling.^116^

Surface charges that vary with pH have a direct impact on floc characteristics and coagulation tendency. According to the findings of a recent study, lower pH enhances the interaction between external organic matter and membrane surface, resulting in increased membrane fouling.^117^ Similarly, the authors also assessed the influence of pH on coagulation; the findings emphasized the impact of pH on isoelectric point (pHiep) of different coagulants, influencing floc size and its formation rate. The isoelectric point of a titanium xerogel coagulant was favored by acidic conditions, allowing the aggregation of algae and organic matter upon dosing.^115^ However, basic conditions were not able to establish charge neutrality and thus the accumulation was lower. The development of mineral foulants and, consequently, the inorganic fouling caused by the precipitation of calcium, phosphorus, and iron increased as the pH linked with algal photosynthesis environment escalates.^117^

Temperature also plays a critical role in AMBR performance. Higher temperature reduces drag forces on the membrane by lowering water viscosity, which in turn increases membrane permeability. In addition, temperature directly affects enzymatic activity, which influences the synthesis of algal organic matter (AOM). In fact, increasing the temperature from 15 to 30 °C causes a decrease in extracellular organic matter (EOM) secretions and thus impacting the overall performance.^115^

### 4.3. Algal biomass

One of the most serious issues faced by AMBRs is the membrane fouling which may occur as a result of growth of algal biomass. Membrane fouling takes place when a membrane gets blocked or covered with particulates, yielding decreased permeability and, consequently, reduction in treatment effectiveness. The fouling capacity of algal biomass is determined by physical and chemical features including surface charge, size, shape, and the presence of extracellular polymeric substances (EP).^118^ The high EP content of algal biomass is responsible for the production of biofilms on the membrane surfaces.^119^ Several techniques have been developed to reduce the accumulation of biomass and membrane fouling in AMBRs. Pre-treatment techniques including coagulation and flocculation are commonly used to eliminate larger particles and colloids prior to their arrival in MBR unit.^120^

### 4.4. Mechanical aeration

Membrane bioreactors find extensive applications in wastewater treatment as this technology is simple to use, takes up very little space, and is capable of eliminating multiple pollutants from wastewater in a single process. However, two issues including membrane fouling and high energy consumption have hampered the AMBRs performance and limit their extensive exploitation. Various studies have been conducted to address membrane fouling by the deployment of optimization of operational settings and the usage of modified polymeric membranes. The high energy consumption of AMBRs is mostly related with aeration which is required for membrane cleaning, aerobic-activated sludge biotreatment, and floc agitation. Additionally, mechanical aeration consumes approximately 55 to 90% of the overall energy supplies of AMBR operation.^121^ As a result, it is necessary to decrease membrane fouling and rate of aeration of AMBR units in order to improve its performance and reduction in energy consumption.^119,122^

### 4.5. Hydraulic retention time and sludge retention time

Both the HRT and the SRT are crucial design and operating parameters because they control the amount of sludge withdrawn from the reactor and the type of microorganisms living there, as well as the biomass concentration and substrate usability in AMBRs. The ability to manage the SRT independently of the HRT is the main benefit of membranes in AMBRs. The volume of sludge to be disposed of in biological systems and the rate of microbial growth are both determined by SRT. AMBRs running on lengthy SRTs offer the production of highly useful microbiological species than those with short SRTs. However, AMBRs running with long SRT result in low biomass wastage along with low N and P removal as these elements are mostly eliminated using the discarded algal biomass. HRT has a significant impact on the effectiveness of the solid–liquid separation and biomass concentration as it directly determines the nutrient loading and treatment capacity of any biological reactor. In order to maximize the creation of algal biomass and to remove N and P from wastewater, it is highly desirable to find the optimal operating SRT/HRT range.^71^

### 4.6. Membrane fouling

Extracellular polymeric substances and microalgae cells have both been recognized as significant contributors towards membrane fouling. A myriad of studies have been devoted to explore the influence of numerous parameters on algal biofouling and separation performance AMBRs.^123^ The potentially affecting parameters may include membrane properties such as pore size and membrane materials, algal species, cell size, metabolic products like protein and carbohydrate fractions, and operational parameters (*i.e.*, HRT and SRT). As a result of the high susceptibility for fouling in algal membrane filtration process, fouling control measures are recognized as critical for their long-term operations. To address this issue, several techniques including chemical and physical cleaning not only reduce the effect of fouling but also extend the life of the membranes. Recent studies report that surface-modified membranes incorporating hydrophilic or photocatalytic coatings (for example, TiO_2_ or graphene oxide) can enhance fouling resistance by limiting extracellular polymeric substance (EP) adhesion. Biological strategies such as quorum-quenching bacteria or enzymes are being explored to disrupt biofilm signaling, while dynamic membranes formed by algal bacterial flocs offer self-regenerating filtration layers.^124^ These emerging approaches collectively show up to 20–40% improvement in flux recovery compared with conventional chemical cleaning alone. However, aforementioned fouling control techniques are highly energy intensive and require bulk of chemicals leading to imbalance the ecosystem.^125^

### 4.7. Inhibitory compounds

Algal and bacterial cells in AMBRs are susceptible to the poisonous and inhibitory substances found in wastewater as toxic substances can inhibit cells growth by hampering their metabolic operations. Industrial discharges and household goods are just two examples of the many potential sources of toxic and inhibitive chemicals contained in wastewater.^126^

### 4.8. Algal–bacterial consortia

For an effective pollutant removal, biomass generation, and membrane fouling control, algae and bacteria must have a harmonious and cooperative relationship. To this end one critical parameter, that can significantly influence the overall performance of AMBR system for wastewater remediation, is the selection of suitable algal–bacterial consortia. Notably, an effective pollutant removal, membrane fouling reduction, and biomass generation, necessitates a well-balanced and synergistic coherence between algae and bacteria present in a treatment system.^127^ To this note, several key factors such as nutrient demands, compatibility degree, growth rates, metabolic capabilities, and capability to endure fluctuating environmental conditions should be kept in mind during the selection of algal and bacterial strains for the consortia.^128^ In the absence of specified guidelines recommended for the choice of an algal–bacterial consortium for AMBRs, it becomes inevitable to explore characteristics of water samples, operational conditions, and treatment objectives. Additionally, the selection of the best combination of bacteria and algae consortium for a defined application can be executed through extensive laboratory-scale studies and keeping an eye on the performance of varied algae and bacteria consortia.^44^ In one such study, an effective microalgae–bacteria consortium was deployed and it was observed that 80 mg L^−1^ of chlortetracycline did not harm a consortium of microalgae and bacteria contrasting to the situation where the pure microalgal culture was able to withstand only 60 mg L^−1^.^129^ Comparing single algae and algal–bacterial consortia in the context of AMBRs involves considering their respective roles, efficiency, and performance within these systems. One key benefit of membrane integration is that the membrane module simultaneously retains biomass and clarifies effluent, eliminating the need for separate harvesting steps such as flocculation or centrifugation used in standalone algal systems. This integration can reduce biomass harvesting costs by up to 30–40% and produce concentrated sludge (1.5–3 g L^−1^) suitable for direct downstream processing.^130^ However, membrane replacement and fouling management add recurring costs, which must be balanced against savings in harvesting and sludge handling.

### 4.9. Nutrient's concentration

Algae growth is strongly dependent on suitable supplies of nitrogen and phosphorus as nutrients however, nutrient removal during the course of water treatment processes *via* AMBRs can limit the nutrient supplies to algae. Consequently, development and activity of algae and bacteria can be impeded by constraints in nutrients supplies, resulting in compromised treatment outcomes. On the other hand, too many nutrients are also detrimental for proper growth of algae and bacterial consortia. The development of algal blooms and excessive algal growth are two unfavorable outcomes from imbalanced nutrient proportions. In addition to reduction in wastewater treatment efficiencies, imbalanced nutrient supplies may end up with several difficulties in terms of elevated membrane fouling, reduced DO, and possible toxins generation.^131^

### 4.10. Reactor design

In order to achieve optimized performance of AMBRs, an appropriate bioreactor design is highly inevitable. The configuration of the reactor can affect the hydrodynamics and mass transfer of pollutants and nutrients. Further, mixing efficiency, membrane fouling, and biomass accumulation can all be impacted by the reactor's size and form. Consequently, improving the reactor design can lower the system's operating and maintenance expenses while increasing the removal efficiency of dangerous and toxic impurities.^114^ Following possible design arrangements for a typical AMBR system can be adopted: (1) External Membrane Bioreactors (EMBR), (2) Submerged Membrane Bioreactors (SMBR), (3) Hybrid Membrane Bioreactors (HMBR), (4) Integrated Membrane Bioreactor (IMBR), (5) Membrane Aerated Biofilm Reactor (MABR), and (6) Moving Bed Bioreactor (MBBR). Among these design arrangements, EMBR and SMBR are most commonly employed designs.^132,133^ A slight difference in the two designs (EMBR and SMBR) lies in the position of the membrane unit which exists outside the bioreactor of EMBR contrasting to SMBR where the membrane unit lies inside the bioreactor.

In any MBR design, it is crucial to employ high aeration intensity to deal with the high non-Newtonian viscosity and satisfy the microbiological oxygen necessity in order to provide air scouring of membranes. However, the high aeration intensity may hinder the activities of the denitrifying and phosphorus-accumulating microorganisms and accelerate the energy consumption, which could end up with less phosphorus and nitrogen removal efficiencies from the system along with high incurred expenses. Undoubtedly, membrane fouling is unavoidable, but periodic cleaning or replacement of the membranes could reduce the overhead costs. Therefore, to address the aforementioned issues, an appropriate MBR design plan must be implemented.^134^

## 5. Performance evaluation of AMBRs

In order to improve life standards and to alleviate worldwide environmental and health concerns from diverse hazardous substances especially persistent organic pollutants (POPs), it is critical to study the precise information regarding their global production and environmental releases. According to a recently published report, as of 2020, a cumulative total of 31.306 million tonnes (mt) of the 25 POPs was manufactured and commercialized globally which resulted in discharge of 20.348 mt into different environmental segments.^7^ Notably, among these globally produced POPs, short-chain chlorinated paraffins were the dominantly produced chemicals with a cumulative of 8.795 mt.^135^

Among the various water treatment technologies, AMBRs demonstrate significant in the elimination of hazardous contaminants from wastewater.^21^ Microalgae exhibit substantial affinity for a wide range of contaminants, including pharmaceuticals, personal care products, heavy metals, and nutrients.^136,137^ Adsorption, biosorption, biodegradation, and bioaccumulation are among the several mechanisms through which AMBRs can potentially eliminate toxicants.

An exponential benefit of AMBRs is their capacity to accomplish the concurrent removal of diverse toxicants.^138,139^ Furthermore, AMBRs can operate at lower HRT compared to conventional MBRs, which reduces space requirements and energy consumption. Additionally, AMBRs are considered environment friendly and workable technologies, as the algal biomass could be benefited for biofuel production and other beneficial purposes.^53^

EPs, also referred to as chemicals of emerging concern, are mostly anthropogenic compounds found in various water bodies, with concentration ranging from microgram to milligram per litre.^140^ These contaminants pose a significant risk not only to human health but also to aquatic ecosystems and other living organisms.^141^ EPs can be classified into organic and inorganic contaminants. Organic pollutants include pharmaceutical compounds, personal care substances, hormones, chemicals from industries, and *etc.* Inorganic pollutants mostly include heavy metals and their compounds.

Concern over the possible harm to human and environmental health posed by a wide variety of contaminants contained in wastewater treatment plants' effluents, which are frequently discharged into the environment, has grown in recent decades. Determining the origins of both current and EPs from the primary waste streams (such as industrial and residential wastewater) may offer important insights into a better comprehension and effective waste management. Among the most commonly detected Eps are pharmaceutical and cosmetic products, perfluorinated compounds (PFCs), gasoline additives, brominated and organophosphate flame retardants, and various nanomaterials. However, only a few studies have looked into the algal-bioremediation strategies in pilot-scale operating conditions.

### 5.1. Removal of pharmaceuticals from wastewater by AMBRs

Micropollutants, also recognized as emerging contaminants, include a wide range of pharmaceutical, pesticides, and PCPs. Agricultural and industrial effluents, personal hygiene products, cosmetics, pharmaceuticals, and hospital streams are only a few of the many sources of these pollutants that are directly linked to human activity.^142^ Among pharmaceuticals, antibiotics represent a major class of contaminants. These chemical substances, which play a vital role in health care by inhibiting and killing microorganisms, have become a growing environmental concern due to their widespread and often excessive use. Antibiotic pollution in aquatic environments is increasingly serve, raising the urgent need for effective removal strategies.^143^ Alarmingly, over 180 000 tons of antibiotics are discharged into the environment annually, with many of these compounds exhibiting high stability, enabling them to pass through conventional treatment processes and accumulate in the environment.^144^

Conventional wastewater treatment methods typically may include physical, chemical, and biological processes such as photodegradation, membrane separation, and advanced oxidation.^145,146^ Recently, microalgae mediated bioremediation has gained scientific attention as an ecologically comprehensive and sustainable strategy for removing antibiotics and other pharmaceutical residues. Microalgae are particularly attractive due to their resilience and adaptability to harsh environments, making them well-suited for the treatment of diverse pollutants.^147^ Additionally, the resulting algal biomass can be repurposed for fuel, fertilizer, and even pharmaceutical applications, reducing the risk of secondary contamination.^148^

**Fig. 9:**
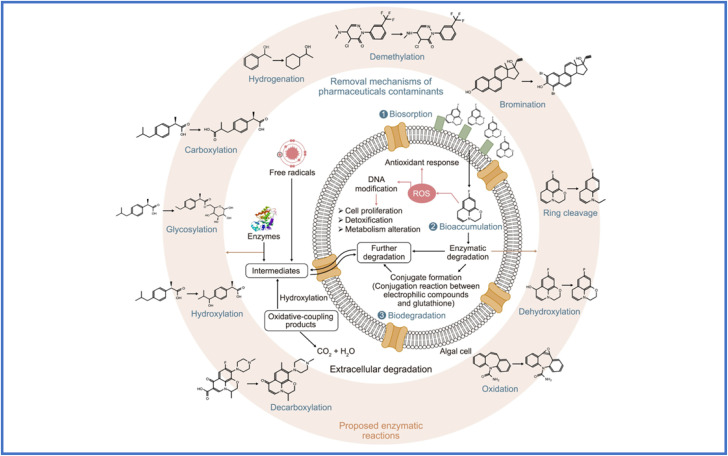
Phycoremediation pathways involved in the removal of pharmaceutical compounds from aqueous solutions using microalgae reproduced from ref. 185 with permission from Elsevier, *Environmental Science and Ecotechnology*, vol. **13**, p. 100205, Copyright 2023.

Phycoremediation mechanisms of pharmaceuticals are highly dependent on the type of target pharmaceutical pollutant, algal species used, and conditions (HRT, SRT, temperature, pH, nutrient dosage and *etc.*) used during the remediation process.^149^ According to the literature, phycoremediation mechanisms of pharma-based pollutants may include biodegradation, sorption, and bioaccumulation as described in Table 1 and Fig. 9.

**Table 1:** Removal of pharmaceuticals, pesticides, and personal care products from aquatic systems using algae^a^

Sr. no	Emerging pollutant class and targets	Nature of water sample	Reactor type	Algal or algal bacterial strains	Optimum pH/temperature (^o^C)/initial concentration (mg L^−1^)/time (days)	Removal (%)	Mechanism of removal	References
**(A) Pharmaceuticals**								
**(a) Antibiotics**								
1	SMX	Synthetic wastewater	abMABR	*Methylophilus*, *Pseudox anthomonas*, and *Acidovorax*	—/25/0.191/1–32	44.6–75.8	Biodegradation	150
2	SMX	Wastewater	HRAP	*Chlorella* sp. *Scenedesmus* sp.	—/—/—/6	95	Biodegradation	151
3	SMX	Wastewater	ABR	*C. protothecoides* and *C. vulgaris*	—/25/0.001/10	77.3	Biodegradation	152
	OFC					43.5		
4	SMX	Synthetic wastewater	PBR	*C. sorokiniana*	—/25/5/10	86.57	Biodegradation	153
5	ERY	Synthetic waste water	AnMBRs	*Haematococcus pluvialis*	7–7.3/25/37.3–100/30	94.41–98.15	Biodegradation	154
	SMX					94.42–98.15		
	TET					69.75–89.73		
6	SMR	Synthetic waste water	AMPBR	*Haematococcus pluvialis*, *Selenastrum capricornutum*, *Scenedesmus quadricauda*, and *C. vulgaris*	—/25/−0.1/0–180	43.28–75.73	Biodegradation	155
	SMX					43.57–75.42		
	SMM					36.91–77.11		
	TMP					15.73–75.24		
	CTM					25.97–94.76		
	AZI					48.91–99.10		
	ROX					39.36–95.40		
	LOM					45.19–86.37		
	LEV					1.40–57.38		
	FLU					15.24–53.57		
7	AZI	Synthetic water	PBR	*Chlamydomonas reinhardtii*	—/25/0.1/14	10–67	Photodegradation	156
	CTM			*C. sorokiniana*		0–36	Sorption	
	ERY			*Dunaliella tertiolecta*		30–33	Biodegradation	
	CFC			*Pseudokirchneriella subcapitata*		51–100		
	OFC					22–88		
	NFC					46–100		
	TMP					11–34		
	SPY					48–93		
	PMA					57–85		
8	SCM	Surface water	PBR	*Phenylobacterium*, Sphingomonadaceae, and Caulobacteraceae	7/23/0.1/8	97	Photodegradation	157
	SMX					98		
9	CPF	Synthetic wastewater	PBR	*C. vulgaris*	7/25/0.32/69.7 h	88	Photo biodegradation	158
	CYP					93.12		
10	SMZ	Synthetic waste water	PBR	*Chaetoceros muelleri* and biochar	—/—/0.311/8.1	64.8	Biodegradation photolysis	84
11	Metronidazole	Waste water	PBR	*Chlorella vulgaris*	9–10/25/5 µM/18–20	100	Adsorption	159
12	SDZ	Marine aquaculture wastewater	BF-MPBR	*C. vulgaris*	7.75/26/0.046–0.14/70	61.0–79.2	Biodegradation	160
	SMZ					50.0–76.7		
	SMX					60.8–82.1		
13	CPF	Synthetic waste water	PBR	*Chlamydomonas* sp. Tai-03	7.2/30/10/6	100	Biodegradation photolysis	161
	SDZ					54.5		
14	SMX	Wastewater treatment effluent	PBR	Mixed consortium of *C. sorokiniana* with bacteria	8.46/21/0.05/7	54.34	Biodegradation	162
15	SMX	Synthetic wastewater	MBR	*C. pyrenoidosa*	—/25/0.4 µM/5	99.3	Biodegradation	163
					Presence of sodium acetate (0–8 mM)			
16	LEV	Synthetic wastewater	PBR	*Chlorella vulgaris*	—/27/1/11	91.5	Bioaccumulation	164
17	TET	Wastewater	MBR	Mixed liquor solids	—/21/	97	Degradation, sorption	165
	4-Epitetracycline					95		
	Doxycycline					90		
	NFC					90		
	CFC					89		
	AZI					78		
	SMX					66		
	OFC					56		
	ERY					12		
**(b) Steroids**								
1	Progesterone	Synthetic waste water	PBR	*Scenedesmus obliquus* & *C. pyrenoidosa*	—/25/5/—	>95	Biotransformation	166
	Norgestrel					40		
2	17 β-Estradiol	Urban wastewater	PBR	*Scenedesmus obliquus* & *Chlorella* sp.	6.18/25/2/0.5	100	Photo biodegradation	167
**(c) Analgesics**								
1	Ibuprofen	Urban wastewater	Semi-closed tubular horizontal PBR	*Green microalgae*	8–10/24–41/8–615 ng L^−1^/—	70	Photodegradation	168
2	TRA	Synthetic waste water	PBR	*Chaetoceros muelleri* and biochar	—/0.332/8.1	69.3	Biodegradation photolysis	84
3	Paracetamol	Synthetic wastewater	PBR	*Chlorella sorokiniana*	7.5/25/250/7–8	67	Biodegradation	169
4	Ibuprofen	Natural wastewater	PBR	*Chlorella* sp. *Scenedesmus* sp.	—/23/0.1/10	99	Biodegradation	170
	Caffeine					99		
5	Acetaminophen	Wastewater	MBR	Mixed liquor solids	—/21/	100	Degradation, sorption	165
	Ibuprofen					100		
	Naproxen					100		
	2-Hydroxy-ibuprofen					100		
	Codeine					99		
	Methylprednisolone					86		
	Caffeine					100		
	Paraxanthine					100		
	Cotinine					98		
**(d) NSAIDS**								
1	DCN	Wastewater	HRAP	*Chlorella* sp. *Scenedesmus* sp	—/—/—/6	71	Biodegradation	151
2	DCN	Agricultural runoff	HTH-PBR	*Pediastrum* sp. *Chlorella* sp. *Scenedesmus* sp. *Gloeothece* sp.	8.3–9.7/9.4–15/—/135	61	Photo biodegradation	80
**(e) Antidepressant**								
1	VFX	Synthetic water	PMBR	*Chlamydomonas reinhardtii*, *C. sorokiniana*, *Dunaliella tertiolecta* and *Pseudokirchneriella subcapitata*	—/25/0.1/14	4–17	Photodegradation	156
							Sorption	
							Biodegradation	
**(f) Antidiabetic**								
1	Metformin	Wastewater	MBR	Mixed liquor solids	—/21/	99	Degradation, sorption	165
**(g) Lipid regulator agents**								
1	Atorvastatin	Wastewater	MBR	Mixed liquor solids	—/21/—/—	99	Degradation, sorption	165
	Gemfibrozil					98		
**(h) Psychiatric drugs**								
1	FLX	Wastewater	HRAP	*Chlorella* sp. *Scenedesmus* sp.	—/—/—/6	66	Biodegradation	151
2	CBZ	Wastewater	HRAP	*Chlorella* sp. *Scenedesmus* sp.	—/—/—/6	32	Biodegradation	151
	LMG					87		
3	Diazepam	Urban wastewater	Semi-closed tubular horizontal PBR	*Green microalgae*	8–10/24–41/8–615 ng L^−1^/—	94	Photodegradation	168
	Lorazepam					83		
	Oxazepam					71		
4	CBZ	Synthetic waste water	PBR	*Chaetoceros muelleri* and biochar	—/—/0.33/8.1	68.9	Biodegradation photolysis	84
5	Amitriptyline	Wastewater	MBR	Mixed liquor solids	—/21/—/—	85	Degradation, sorption	165
	Paroxetine					82		
	Diazepam					54		
	FLX					35		
	CBZ					28		
	Alprazolam					21		
**(i) Beta blockers**								
1	MET	Wastewater	HRAP	*Chlorella* sp. *Scenedesmus* sp.	—/—/—/6	65	Biodegradation	151

**(B) Pesticides**								
**(a) Herbicides**								
1	ATZ	Surface water	PBR	Immobilized *Citricoccus* sp. strain *C. vulgaris*	5/25/50/2	100	Biodegradation	171
2	Propanil	Surface water	PBR	*Scenedesmus* sp. and *Chlorella* sp.	8.1–8.4/25/0.05/8	99	Biodegradation	172
3	BMC	Surface water	PBR	*Phenylobacterium*, Sphingomonadaceae, and Caulobacteraceae	7/23/0.1/8	99	Photodegradation	157
	ATZ					98		
4	ATZ	Synthetic water	PBR	*Chlorella* sp.	—/25/0.004/8	83	Photo biodegradation	173
5	ATZ	Synthetic wastewater	HMPBR	Microalgae and bacteria	6.8–7.2/25/0.01/12 h	95.39	Photo biodegradation	174
6	ATZ	Synthetic ground water	PBR	*Scenedesmus* sp. immobilized beads	—/20/0.09–0.1/10–29	70	Photo biodegradation	175
	Oxadiazon					100		
	Triallate					100		
**(b) Insecticides**								
1	THIA	Wastewater	PBR	*Scenedesmus* sp.	—/25/60/12	100	Degradation	176
2	Acetamiprid	Surface water	PBR	*Scenedesmus* sp. and *Chlorella* sp.	8.1–8.4/25/0.005/8	71	Biodegradation	172
3	CPF	Synthetic wastewater	PBR	*C. vulgaris*	7/25/0.32/69.7 h	88	Photo biodegradation	158
	CYP					93.12		
4	Imidacloprid	Synthetic wastewater	PBR	*Nannochloropsis* sp.	8/25/–/7	52.5	Adsorption	177
							Biodegradation	
5	Alachlor	Synthetic wastewater	Semi-closed tubular horizontal PBR	Microalgae/bacteria consortium	8.3/24.2//—/5	100	Photodegradation biodegradation	178
	Azinphosethyl					100		
	Chlor-fenvinphos					100		
	Desisopropil					100		
	Atrazine					100		
	Fenthion oxon					100		
	Fenthion sulfoxide					100		
	Irgarol					100		
	Linuron					100		
	Malaoxon					100		
	Ter-butylazine					100		
	MCPA					88		
6	Diazinon	Synthetic water	MBR	*C. vulgaris*	—/—/20/12	94	Biodegradation	179

**(C) Personal care products**								
1	Methylparaben	Synthetic waste water	PBR	*Acinetobacter calcoaceticus*	7.5/25/0.8/7	>50	Photodegradation	180
				*Chlorella vulgaris*				
2	Methyl paraben	Synthetic waste water	AFCMBR	*Syntrophorhabdus* and *Longilinea*	—/—/1/30 at HRT of 16 h	99	Biodegradation	108
							Biosorption	
3	Methyl paraben	Synthetic wastewater	PBR	*Chlorella vulgaris*	7.5/25/0.796/7	33.16	Photodegradation	181
4	Triclosan	Grey water	PBR	*Nannochloris* sp.		99	Photobiodegradation	182
	TMP					10		
5	Triclosan	PCP rich grey water	SMBR	*C. vulgaris*	7/20–27/—/16 h	98.20	Biodegradation	105
	Methyl paraben					99.96		
	Propylparaben					99.97		
	Ethyl paraben					64.28		
	Butyl paraben					75		
	2-Phenoxyethanol					99.99		
6	Triclosan	Seawater	MBR	*Phaeodactylum tricornutum*	6/25/1/3 h	100	Biodegradation	183
							Biosorption	
7	Tonalide	Agricultural runoff	HTH-PBR	*Pediastrum* sp. *Chlorella* sp. *Scenedesmus* sp. *Gloeothece* sp.	8.3–9.7/9.4–15/—/135	73	Photo biodegradation	80
	Galaxolide					68		
8	Triclosan	PCP rich grey water	PBR	*Nannochloris* sp.	7.8/25/—/7	100	Adsorption	184
							Photolysis	
9	Triclosan	Wastewater	MBR	Mixed liquor solids	—/21/—/—	99	Degradation, sorption	165
	Miconazole					94		
	Triclocarban					92		
	Enalapril					99		
	Furosemide					99		
	Atenolol					77		
	Diltiazem					73		

a Azithromycin (AZI), Clarithromycin (CTM), Erythromycin (ERY), Ciprofloxacin (CFC), Ofloxacin (OFC), Trimethoprim (TMP), Sulfapyridine (SPY), Sulfadiazine (SDZ), Sulfamethazine (SMZ), Norfloxacin (NFC), Pyridopyrimidine (PMA), Venlafaxine (VFX), Sulfamethoxazole (SMX), Tetracycline (TET), Sulfamerazine (SMR), Sulfamonomethoxine (SMM), Roxithromycin (ROX), Lomefloxacin (LOM), Levofloxacin (LEV), Flumequine (FLU), Sulfacetamide (SCM), Lamotrigine (LMG), Metoprolol (MET), Fluoxetine (FLX), Diclofenac (DCN), Bromacil (BMC), Atrazine (ATZ), Chlorpyriphos (CPF), Cypermethrin (CYP), Thiamethoxam (THIA), diethyltoluamide (DEET), Diethylphthalate (DEP), Jialemusk (HHCB), Tuinamusk (AHTN), Ethylhexylmethoxycinnamate (EHMC), Photobioreactor (PBR), Microalgae biofilm membrane photobioreactor (BF-MPBR), Anaerobic membrane reactors (AnMBRs), Algal membrane photobioreactor (AMPBR), Hybrid microalgal–bactrial membrane photobioreactor (HMPBR), Submerged membrane bioreactor (SMBR), Anaerobic = ceramic membrane bioreactor (AFCMBR), High performance liquid chromatography coupled to high-resolution mass spectrometry (HPLC-HRMS), High-rate algae-bacteria pond (HRAP), Algal–bacterial membrane aerated biofilm reactor (abMABR), UPLC coupled to a time-of-flight mass spectrometry in negative ionization mode with an electrospray ionization (ESI^−^) source (UPLC-QTOF-MS), Semi-closed (hybrid) tubular horizontal photobioreactor (HTH-PBR).

Recently, a microalgae; *Haematococcus pluvialis*, a freshwater species of *Chlorophyta* capable to form large quantities of astaxanthin, has been bioaugmented into an aerobic AMBR to explore its capacity to treat 3 most common occurring antibiotics including sulfamethoxazole (SMX), tetracycline (TET) and erythromycin (ERY) in wastewater, lowering membrane biofouling, and effects on composition of microbial communities. The study achieved a maximum removal efficiency of 89.73% for TET, with a 33% reduction in membrane biofouling.^154^ Noteworthy, complex mixtures of pollutants in wastewater could cause difficulties in their complete elimination and may involve diverse mechanisms (sorption, photodegradation, membrane rejection, abiotic, bioaccumulation, and biodegradation) of their removal. In one such attempt to investigate the insights into the removal mechanism of a mixture of 9 antibiotics (3 fluoroquinolones: ciprofloxacin, ofloxacin, norfloxacin; 3 macrolides: azithromycin, clarithromycin, ERY, and three different classes of antibiotics including pipemidic acid, trimethoprim, and sulfapyridine) and 1 antidepressant (venlafaxine), 4 strains of microalgae (*Chlamydomonas reinhardtii*, *Chlorella sorokiniana*, *Dunaliella tertiolecta*, and *Pseudokirchneriella subcapitata*) under different experimental conditions were employed.^156^ Results showed that photodegradation was the dominant removal mechanism for ciprofloxacin, ofloxacin, norfloxacin, and pipemidic acid (>78%), while a combination of sorption and biodegradation was responsible for removing for total removal of azithromycin, clarithromycin, and ERY. However, for sulfapyridine elimination mechanism was purely algal biodegradation as other two mechanisms including sorption and photodegradation exhibited least efficiencies. From these findings, it can be inferred that pollutant removal significantly depends on the algal strains and nature of pollutant. However, most stable (persistent) pollutants would require harsh conditions for their complete removal. Another study evaluated the removal pathway of 10 mixed antibiotics along with nutrients deployed four freshwater microalgae strains (*Haematococcus pluvialis*, *Selenastrum capricornutum*, *Scenedesmus quadricauda*, and *Chlorella vulgaris*) in MPBRs in a continuous flow mode at lab-scale. It was observed that biodegradation was the major removal mechanism of the antibiotics in *Haematococcus pluvialis* MPBR, with excellent removal efficiencies (53.57–96.33%). However, bioadsorption, bioaccumulation, membrane rejection, and abiotic contributed minor in antibiotics removal mechanism.^155^ Likewise, Xie *et al.* demonstrated that *Chlamydomonas* sp. (Tai-03) was efficiently capable to remove antibiotics through biodegradation (65%) and photolysis (35%).^161^ Since ciprofloxacin is more easily adsorbed onto biomass than sulfadiazine, they noted that adsorption might be crucial in fostering biodegradation. Despite of several serious efforts conducted for the removal of pharmaceuticals, some of these pharmaceuticals are of recalcitrant nature and can pass through several stages of purification. For instance, pharmaceuticals such as carbamazepine, limited biodegradation is often linked to the absence or low activity of key oxidative enzymes such as laccase, peroxidase, and dioxygenase in algal bacterial consortia.^186^ These enzymes catalyze aromatic ring cleavage and hydroxylation, which are necessary for complete mineralization. In most AMBR systems, carbamazepine undergoes only partial oxidation to stable intermediates because of low enzyme affinity and restricted co-metabolism, highlighting a fundamental biochemical bottleneck in algal-mediated degradation. Additionally, above mentioned recalcitrant pharmaceuticals may accumulate in different environment segments leading to their entrance in food chain which may consequently pose serious threats to human kind and other living organisms.

### 5.2. Removal of pesticides from wastewater by AMBRs

Pesticides, a wide range of heterogenous compounds (*e.g.*, insecticides, herbicides on lawns, fungicides, algicides in paints and coatings, and roof-protection agents in sealants), have historically been extensively deployed in crops protection against the unwanted microorganism. However, their uncontrolled and excessive application can lead to microbial resistance and their released into various environmental compartments, resulting in ecological and human health risks (especially when they enter the food web). These contaminates are recognized as major hazardous substances of various waterways and their uncontrolled exposure generates microbial resistance and can lead to enter in the food chain of organisms living in terrestrial and aquatic habitats. Furthermore, untreated water effluents from pesticide industries are also considered the major contributory towards pesticide contamination.

Diverse traditional wastewater treatment technologies, such as activated sludge, moving bed biofilm reactors, trickling filters, microalgae, nitrification, and fungi, and bacteria treatments, as well as biological activated carbon, rely on biological activities and decomposition as the primary elimination approaches.^187^ Further improvement in their performance in terms of complete removal/mineralization of targets including pesticides is highly desirable and can be augmented in conjunction with other biologically active processes to boost pesticide removal. Among several technologies employed for the pesticides removal in water, utilization of algal biomass has received great attention due to their multiple advantages in terms of simultaneous pesticide-containing wastewater treatment and nutrient recovery for microalgae growth along with minimum toxic sludge production.^188^ Moreover, the role of algae is not only to serve as a biofilter but also to transform the target pesticides into less toxic metabolites as microalgae utilize pesticides as their carbon and nitrogen sources. The elimination of pesticides through microalgae generally occurs through biosorption, bioaccumulation and biodegradation however, the removal efficiency greatly depends on the lipid content, strain, and the chemical structure of the pesticide.^189^ For instance, among the four investigated species (*Scenedesmus obliquus*, *Chlamydomonas mexicana*, *Chlorella vulgaris*, and *Chlamydomonas pitschmannii*) *Chlorella vulgaris* has been found to assimilate 94% at significantly high concentration (20 mg L^−1^) of diazinon, a toxic insecticide, and then transform into a less toxic metabolite (2-isopropyl-6-methyl-4-pyrimidinol).^179^ However, it was demonstrated that further rise in diazinon concentration to 40 mg L^−1^ significantly resulted in >30% growth inhibition of *Chlorella vulgaris*.

It is also in observation that the immobilization technology, an emerging approach in bioremediation, relies on controlled placement of free microorganisms in a determined geographic area using physical or chemical strategies to keep them viable and active.^190^ Nonetheless, this technique mostly offers best performance in terms of removal efficiencies of pesticides relative to free cells which may be attributed to a context of high population density with a low volume.^191^ Furthermore, immobilization of biomass can be utilized multiple times and it enables cell storage for extended periods without impairing degradability thus making it economically viable approach. In an attempt to access the performance of immobilization approach relative to free cells in water samples containing two pesticides including chlorpyriphos and cypermethrin, two photobioreactors, including biochar (acting as substrate to immobilize algae) and *Chlorella vulgaris* (reactor 1), and *Chlorella vulgaris*/activated sludge (reactor 2) were employed.^191^ The evaluation of data through response surface methods indicated that phycoremediation system containing immobilized *Chlorella vulgaris* enabled abatement of pesticides 88–93% at 69.7 h contact time and 0.32 mg L^−1^ initial concentration of targets. Another group of researchers co-immobilized *Chlorella vulgaris* and *Citricoccus* sp. strain TT3 consortium in porous beads to investigate degradation of atrazine.^171^ Higher than 40% atrazine abatement was achieved under optimized conditions which reflected the positive impact of immobilization of algal biomass. Interestingly, slight modifications in AMBRs and/or attachment of useful additional accessories may result in further enhancement in phycoremediation efficiencies. Recently, in one such study, removal of two pesticides (atrazine and bromacil) in groundwater was investigated through a photobioreactor containing immobilized microalgae (*Phenylobacterium*, Sphingomonadaceae, and Caulobacteraceae) and bacteria consortium in polyurethane foam followed by a cork filter (CF).^157^ Pesticide transformation products were identified through gene-based metataxonomic assessment, supporting biodegradation as the main contributing mechanism. The modified PBR-CF protocol enabled pesticides removal efficiency of 95% at an HRT of 8 days, however, it was observed that pesticide removal efficiency was strongly dependent on HRT. With shorter HRT, removal efficiency significantly reduced from 95% at an HRT of 8 days to 23–45% at an HRT of 2 days. A comprehensive illustration for the performance of AMBRs in context to pesticides removal is given in Table 1.

### 5.3. Removal of personal care products from wastewater by AMBRs

To accommodate the increasing demands for improved human health standards globally, several personal care products (PCPs) are being used in amounts comparable to agrochemicals. These compounds are unconsciously discharged into the environment from both point and nonpoint sources, remain often unmonitored and unregulated.^192^ PCPs are an underestimated group of EPs, with some of these PCPs are enumerated by the United States Environmental Protection Agency and Stockholm Convention as priority pollutants.^193^ Furthermore, they have become environmentally pervasive in all facets of ecosystem due to their wide usage, difficulty in complete degradation due to complicated structures, and inappropriate removal from ecosystem and are attracting significant attention of researchers. Therefore, it is highly desired to efficiently remove these hazardous compounds from water sources (Table 1). To this note, a myriad of research studies has been conducted for the removal of PCPs in different water samples, ranging from synthetic to real-world water matrices. Recently, a group of researchers deployed a SMBR system and achieved removal efficiencies of 98.20%, 99.96% and 99.97% for triclosan, methylparaben and propylparaben, respectively, with their highest concentrations in as prepared PCPs-rich greywater.^105^ They demonstrated that HRT had a striking influence on performance of the SMBR in removing PCP contaminants.

**Fig. 10:**
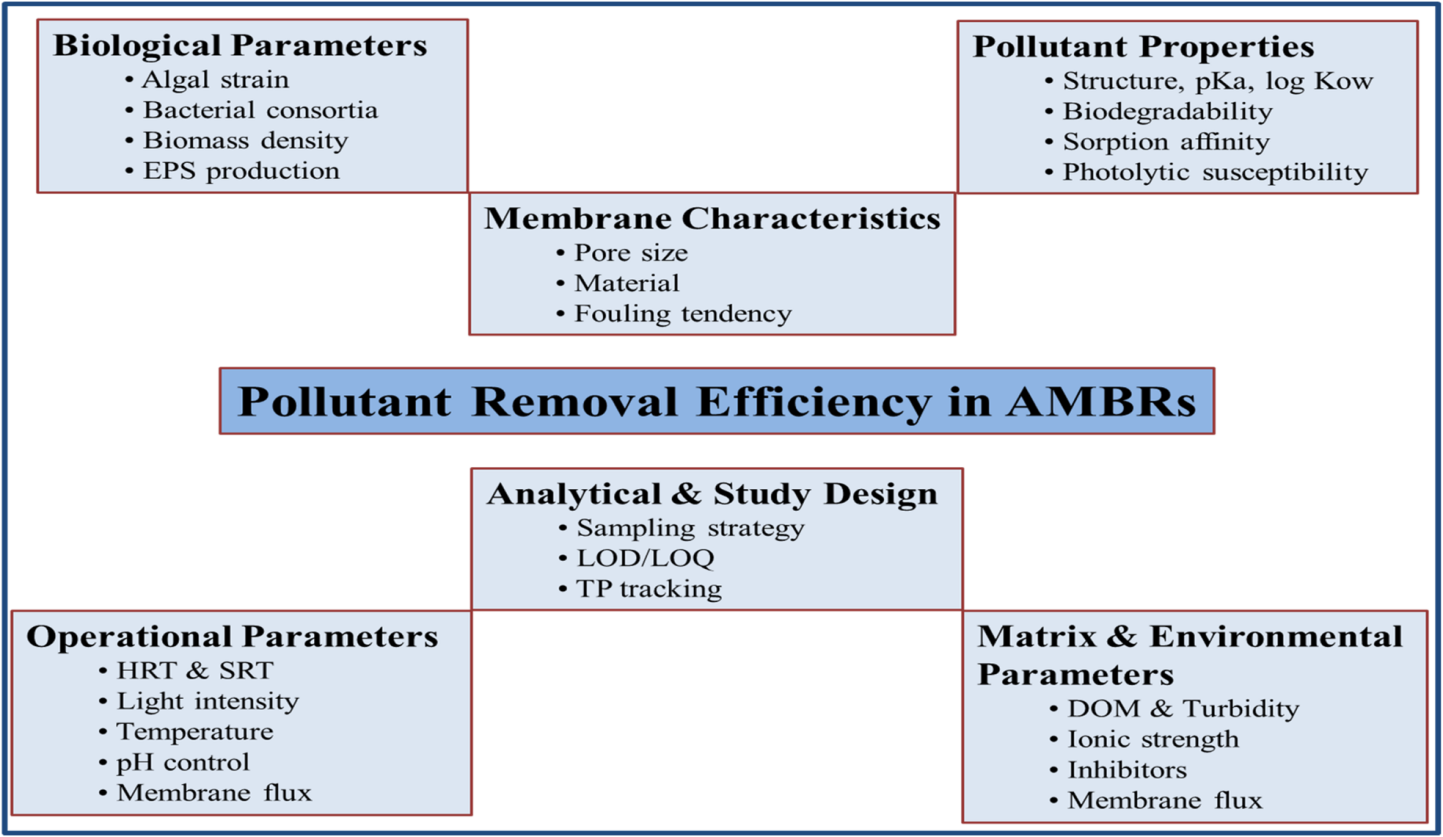
Parameters potentially affecting the removal efficiencies of emerging pollutants through AMBRs.

In AMBRs, the dual mechanism of sorption and biological degradation system enables them to successfully remove targets. The membrane system restricts the movement of high molecular weight targets at the surface, leading to their biodegradation and physical retention.^187^ Recently, in an attempt to compare the performance of different systems, recirculating AMBRs consisting of an anoxic tank, and aerobic tank were employed to investigate the removal of five micropollutants including triclosan from wastewater.^194^ The results revealed that triclosan was completely adsorbed by both anoxic and aerobic sludge. However, in synthetic water, triclosan removal was slightly lower than in real wastewater, likely due to microbial diversity and lower levels of suspended solids, which results in decrease removal rate of triclosan. Generally, the deployment of bacterial and algal consortia results in enhanced bioremediation performance. For instance, in the wastewater treatment of methylparaben, a consortium of *Acinetobacter calcoaceticus* and the microalga *Chlorella vulgaris* achieved removal efficiencies of 77 to 83%, compared to only 30% when using microalgae alone.^180^ Further improvements in PCPs removal can be escalated by the deployment of appropriate bioreactor configurations with optimized experimental conditions and cocultured microalgae with best symbiotic relationships for a specific target. A group of researchers used an AMBR for the removal of multi-compounds including acetaminophen, caffeine, metformin, 2-hydroxy-ibuprofen, ibuprofen, naproxen, clarithromycin, atenolol, carbamazepine, trimethoprim triclosan, ciprofloxacin, norfloxacin, triclocarban, ofloxacin, and paraxanthine from different aqueous streams of a wastewater plant.^165^ They showed that pharmaceutical and PCP removal varied from 34% to >99%. Owing to deposition/cake development and pore clogging by rejected species on the membrane surface, the AMBR's performance was found to decline with filtering time. A conceptual diagram representing diverse parameters that can potentially impact the overall degradation efficiencies of emerging pollutants through the deployment of AMBRs is given in Fig. 10.

### 5.4. Removal of heavy metals removal from wastewater by AMBRs

Noteworthy, it is pertinent to mention that metals are vital for photosynthesis and other metabolic pathways in microalgae; however, their occurrence at higher concentrations can have adverse impacts on the ecosystem.^195^ Heavy metal ions in water streams from diverse sources is alarmingly increasing and it raises severe concerns to biosphere and necessitates their complete assessment and removal.^196^ A myriad of strategies have been employed to eliminate heavy metal ions in aquatic systems, but each exhibits its own pros and cons. Mostly, these approaches necessitate high installation and maintenance costs, as well as operational expenses, and often produce secondary pollutants.^185,197^ Therefore, it is highly inevitable to investigate and deploy robust, eco-friendly, and economically viable approaches.

To this end, microalgae have been recognized for their significant potential in wastewater treatment due to their ability to uptake heavy metal ions and their toxic derivative compounds through biosorption and bioaccumulation mechanisms, as presented in Fig. 10a. The presence of a variety of functional groups, such as deprotonated carboxyl and sulfate groups, as well as monomeric alcoholic groups in microalgae, plays a key role in stimulating of biosorption of heavy metal ions.^198^ Furthermore, extracellular polymeric moieties obtained from microalgae can speed up the overall heavy metal ion sorption but their efficiency is greatly dependent on several other parameters such as nature of heavy metal ion, and operational conditions.^199^ Recently, two acid tolerant microalgae species *Desmodesmus* sp. and *Heterochlorella* sp. were investigated for the simultaneous removal of Cu, Fe, Mn, and Zn from their growing environment at pH 3.5.^200^*Desmodesmus* sp. was especially efficient at removing Fe (up to 86% after 16 days). Whereas, *Heterochlorella* sp. was more efficient at removing Mn, with an adsorption percentage of 84% at 10 mg L^−1^ initial concentration. The cellular analysis confirmed that the removal of the investigated ions occurred primarily through adsorption and uptake, with up to 99% of the ions accumulated inside the cell. In another study, Rajalakshmi *et al.* investigated the potential of *Chlorella* sp. isolated from Yercaud lake for the removal of seven heavy metals, including Cr, Pb, Ni, Cd, Co, Zn, and Cu present in tannery effluent using a small scale photo bioreactor treatment approach.^201^ Accordingly, a significant reduction in the heavy metals content in the tannery effluent after the wastewater treatment was noticed. The maximum uptake efficiency of *Chlorella* sp. for the metals investigated was found to be 95.59, 94.12, 93.94, 93.98, 93.43, 93.84, and 89.38% for Cr, Co, Ni, Cd, Pb, Zn, and Cu, respectively. Furthermore, it was pointed out that the removal mechanism of heavy metals was purely biosorption. To further enhance heavy metal removal efficiencies in AMBRs, the use of dynamic membranes (DM) can be highly beneficial. DMs perform dual function: (a) reduction in membrane biofouling and (b) enhanced heavy metal elimination.^185^ DMs can be easily formed over a polymeric membrane or a mesh membrane bed and can also be removed easily by washing in the reverse direction of water. Furthermore, owing to facile usability and recoverability of microalgae and its non-living mass, DMs are practically feasible approach to be utilized particularly for mercury removal in dental units.^202^

A number of researches have been conducted to evaluate the performance of DMs based AMBRs contrasting to controlled AMBRs for the elimination of heavy metals. In one such attempt Hg removal from dental wastewater (DWW) using microalgae dynamic membrane of *Chlorella vulgaris* suspended particles in a dynamic membrane bioreactor (DMBR) using synthetic DWW has been reported.^203^ The authors compared its performance with a control membrane bioreactor (CMBR) under similar optimized experimental parameters. From the results, it was observed that DMBR outperformed CMBR for Hg removal and was not limited to DWW but can be effectively deployed for effluents with high load of Hg. However, it was noticed that the performance of DMBR in the presence of activated sludge dropped from 85.88 to 79.02% probably because of covering of DM.

Phycoremediation of heavy metals has also been recognized to be affected by the cultivation methods.^204^ To address this issue, three different algal strains/consortia; *Chlorella pyrenoidosa*, *Chlorella phormidium*, and a consortium from Hauz Khas Lake were cultured in suspension and attached biofilm systems for the remediation of individual and multiple heavy metals (*e.g.*, Cd, Cr, Pb, Cu, and Zn) in batch experiments (HRT-6 days).^205^ The authors analyzed biomass production and metal removal and demonstrated that consortia of *Chlorella pyrenoidosa* and the Hauz Khas lake consortium performed better in suspensions systems for individual heavy metals, while *Chlorella phormidium* can perform exceptionally well for variety of effluents containing mixed metals in attached biofilm-based systems.

In another study, researchers evaluated the competitive biosorption of Pb^2+^, Cd^2+^, Cu^2+^, and As^3+^ ions by using native algae in a batch reactor.^206^ They obtained equilibrium data for adsorption of single, binary, ternary, and quaternary metal ion solutions. The removal mechanism was biosorption, which relied on ion exchange with light metal ions such as Na, Ca, and Mg. The removal efficiency of heavy metal ions was found to be greatly influenced by the affinity between the microalgal strains and the heavy metal ions. For instance, Pb^2+^ caused a greater change in the functional groups of algal biomasses due to its high affinity for Pb^2+^. The affinity constants for single metal system followed the sequence: *K*_Pb_ > *K*_Cu_ > *K*_Cd_ > *K*_As_; however, these values reduced in binary, ternary, and quaternary systems. Furthermore, kinetic data revealed that the biosorption of the heavy metal ions followed pseudo-second-order kinetics. This suggests that the specific removal of heavy metal ions by a typical microalgal strains can be related to the presence of specific extracellular polymeric substances. For instance, a low pH enhances the ability of extracellular polymeric substances in *Nostoc linckia* to absorb heavy metal ions (*e.g.*, Co^2+^ and Cr^4+^) due to the presence of negatively charged functional groups.^207^ Based on these findings, it can be concluded that the microalgal affinity for specific heavy metal ions and its capacity to capture can be assessed by evaluating the chemical structure of target metal ions.^185^Table 2 summarizes the remediation of majority of heavy metals ions by microalgae.

Removal of heavy metals, nitrogen, and phosphorous from aquatic systems using algae^a^

**Table:** 

(A) Heavy metals								
Sr. no.	Metals	Nature of water sample	Reactor type	Algal or algal bacterial strains	Optimum pH/temperature (^o^C)/time (days)/initial concentration (mg L^−1^)	Removal (%)	Mechanism of removal	References
1	Cr	Wastewater	MBR	*Anabaena* sp.	32–5/25 ± 2/7/0–20	98	Adsorption	208
2	Zn	Textile wastewater	MBBR-MBR	Mixed strains	7.2–7.3/25/50/	96	Adsorption	97
	Pb				24	92		
	Cr				1.50	85		
	Fe				1.86	88		
					82.4			
3	Pb	Wastewater	MBBR	Mixed strains	12/21/45/20	85	Biosorption	209
4	Cr	Synthetic groundwater	Immersed microalgae MBR	*Chlorella vulgaris*	5–7/—/180/—	32	Adsorption	210
	Cu				2.17	93		
	Ni				0.61	97		
					1.25			
5	Cu	Synthetic wastewater	PBR	*Chlorella* spp. and *Scenedesmus* spp.	—/—/—/1	99.6	Sorption	211
	Zn					97.8		
	Cd					96.4		
	Ni					80.3		
	Cr					12.4		
6	Cr	Tannery effluents	PBR	*Chlorella* sp	7/18–23/20/	95.59	Biosorption	201
	Cu				247.89	89.38		
	Pb				100.89	93.43		
	Zn				190.90	93.84		
					187.67			
7	Ni	Industrial wastewater	RAB	Indigenous microalgae consortium	5.0/25/21/5000/—	>90	Adsorption	212
8	Cd	Lake water	AMBR	*Phormidium (PA6)*	7.05–9.35/25 ± 2/15	95	Biosorption	205
	Cr				1	28		
	Pb				1	80		
	Cu				1	74		
	Ni				1	96		
	Zn				1	98		
					1			
9	As	Acid mine drainage	Sulfidogenic anaerobic MBR	*Desulfovibrio*-like bacteria	3.5–4/35 ± 2/0–48/2.5	99	Adsorption	213
10	Mg	Synthetic ground water	PBR	*Scenedesmus* sp. Immobilized beads	—/20/—/29	100	Adsorption	175
	Zn					92		
	Fe					71		
11	Cu	Tannery effluent	PBR	*Desmodesmus* sp. *MAS1 Heterochlorella* sp. *MAS3*	3.5/23 ± 1/16/	43	Adsorption	200
	Fe				0.5	86		
	Mn				20	32–61		
	Zn				20	84.8		
					10			
12	Cd	Synthetic water	PBR	Immobilized *Chlorella* sp.	6.0/–/10/1	92.45	Biosorption	214
13	Cu	Synthetic wastewater	Spiral tubular bioreactor	Biofilms of mixed consortium	7.9/30/2/4.5	99	Biosorption	215
14	Hg	Dental wastewater	DMBR	*Chlorella Vulgaris*	—/30–50/30/0.2–0.8	85.88	Adsorption	203
15	Pb	Real wastewater	PBR	*Oscillatoria princeps*, *Chlorophyta*	3–5/25/4 h/50	90	Biosorption	216
	Cd							
	Cu							
	As							

a Total nitrogen (TN), Total phosphorous (TP), Microwave plasma atomic emission spectroscopy (MP-AES), Microalgal-based iron nanoparticles (ME-nFe), Inductively Coupled Plasma-Optical Emission Spectroscopy (ICP-OES), Microwave Plasma Atomic Emission Spectroscopy (MP-AES), Moving bed bioreactor membrane bioreactor (MBBR-MBR), Revolving algal biofilm reactor (RAB), Dynamic membrane bioreactor (DMBR), Membrane bed biofilm reactor (MBBR), Electro algae-activated sludge membrane bioreactor (e-AAS-MBR), Microalgal-activated sludge membrane bioreactor (MAS-MBR), Algal membrane photobioreactor (AMPBR), Suspended-solid phase photobioreactor (ssPBR), Microalgal–bacterial granular sludge-marimo (MBGS-MA).

**Table:** 

(B) Nutrients								
Sr. no.	Ions	Nature of water sample	Reactor type	Algal or algal bacterial strains	Optimum pH/temperature/(^o^C)/time in days initial concentration (mg L^−1^)	Removal (%)	Mechanism of removal	Reference
1	NH_4_^+^–N	Synthetic waste water	abMABR	*Methylophilus*, *Pseudox anthomonas*, and *Acidovorax*	—/25/62.4/1–32	92.1	Assimilation	150
2	NO_3_^−^	Agricultural wastewater	PBR	*C. vulgaris*	7/25/1/	88.4	Adsorption	217
	PO_4_^3−^				25 & 4.54	53.74		
3	TN	Domestic wastewater	ABR	*C. vulgaris* NIES-227	7.82/25/14/—	97.2	Assimilation	218
	TP				8.9	100		
					0.8			
4	TN	Dairy wastewater	PBR	*C. vulgaris*	7.45/27/16/98 & 31	87.7	Assimilation	219
	TP					93.5		
5	TN	Synthetic wastewater	ssPBR	*Scenedesmus* sp. *LX1*	7–8/20/1–6/	96	Assimilation	220
	TP				15 & 0.5	98		
6	NH_4_^+^–N	Synthetic wastewater	PBR	Algae bacteria consortium	7.75/18/20/	66–84	Assimilation	221
	P				30 & 5	95–97		
7	TN	Real wastewater	MBGS-MA	Microalgal bacterial consortium	7.5/20/10/	83.4	Assimilation	222
	NH_4_^+^–N				4 & 0.8	94		
8	TN & TP	Synthetic wastewater	PBR	*C. vulgaris*	7/21/—/203 & 285	90	Assimilation	223
9	NO_3_–N	Wastewater	MPBR	*Spirulina* sp.	8.5 and ambient temperature/60–80/—	39.3–40.9	Assimilation	224
	PO_4_–P					43.8–46.6		
10	NO_3_–N	Synthetic waste water	AMPBR	*Haematococcus pluvialis*, *Selenastrum capricornutum*, *Scenedesmus quadricauda*, and *C. vulgaris*	—/25/−0.1/72/0–180	78.03–96.01	Assimilation	155
	PO_4_–P					59.74–100		
11	Nitrate	Surface water	PBR	*Phenylobacterium*, Sphingomonadaceae, and Caulobacteraceae	7/23/180/0.1&8	58	Assimilation	157
	Nitrite					89		
12	TP	Treated municipal water	PBR	*C. vulgaris*	7.4/20/–/9	86.2	Accumulation	225
	TN					81.8		
13	NH_3_	Municipal wastewater	MAS-MBR	*C. vulgaris*	7/25–28/14/—	94.36	Assimilation	93
	P					88.37		
14	TN	Synthetic greywater	MBR	*Scenedesmus*	7.1–8.9/29/—/4–21 & 0.1–100	52	Assimilation	226
	TP					36		
15	NH_3_	Urban wastewater	MBR	*Scenedesmus* sp.	7.9/19.3/—/—	99	Assimilation	227
16	TN	Synthetic wastewater	MBR	*Scenedesmus*	8.2–8.4/27.1/—/10.4 & 6.6	59.5	Assimilation	114
	TP					34.5		
17	NH_3_–N	Municipal wastewater	e-AAS-MBR	*C. vulgaris*	7.29 ± 0.31/25/30/77.6 TN	43.89	Assimilation	68
	PO_4_^3−^–P				13.4	65.60		
18	NH_4_^+^–N	Synthetic wastewater	Semiclosed tubular horizontal PBR	*Nannochloropsis* sp.	8.3/24.2/5/4.4 ± 1.5, 9.3 ± 1.8 &1.6 ± 1.0	93.2	Assimilation	178
	NO_3_^−^–N					53.8		
	PO_4_^3−^–P					100		
19	NO_3_–N	Synthetic ground water	PBR	*Scenedesmus* sp. immobilized beads	—/20/8.8/29	97	Assimilation	175
	TP					99.9		
20	TN	Synthetic wastewater	HMPBR	Microalgae and bacteria	6.8–7.2/25/150/5 & 1	99.64	Assimilation	174
	TP					98.02		
21	NH_4_^+^–N	Municipal wastewater	Hybrid aerobic MBR	*C. vulgaris*	7–8/24/10/	73.6	Assimilation	228
	NO_3_^−^–N				40, 10 & 5	13.4		
	PO_4_^3−^–P					100		
22	TN & TP	Synthetic wastewater	PBR	*C. vulgaris*/*Pseudomonas putida*	7–9/25/1–8	80 & 60–70	Assimilation	90
					50 & 10			
23	TN & TP	Municipal wastewater	PBR	*C. microporum*/wastewater bacteria	7.3–8.5/37/1–12/	88 & 89	Assimilation	229
					39.5 & 5.3			
24	TN & TP	Municipal wastewater	PBR	*C. vulgaris*/wastewater bacteria	9–11/37/7/	24 & 70	Assimilation	230
					141 & 178			
25	TN	Synthetic wastewater	Chemostat bioreactor	*C. vulgaris*/*A. brasilense*	7/32/—/191&258.9	91	Accumulation	231
	T P					75		
26	NH^+4^ & TP	Synthetic wastewater	PBR	*C. vulgaris*/*B. licheniformis*	3.5–7/25/6/20 & 4	86 & 93	Assimilation	232

### 5.5. Nutrient's removal from wastewater by AMBRs

It is well known that the wastewater from the domestic sources generally contains organic contaminants along with nutrients, including nitrogen and phosphorus-containing compounds, which can lead to eutrophication.^44^ Current water treatment technologies for removing nitrogenous and phosphorous compounds mainly rely on separation processes, including electrochemical reduction, activated carbon adsorption, advanced oxidation processes, ion exchange, electrodialysis, and reverse osmosis. However, these methods face several challenges, such as high installation and operational costs, as well as the generation of concentrated brine, which further augment expenses incurred for additional treatment. In contrast, biological processes based on heterotrophic microorganisms are preferred owing to their low costs and generation of harmless gases such as nitrogen. However, they also necessitate the availability of labile organic carbon to donate electrons for accelerated microbial grow and to take up nitrate electron acceptance.

**Fig. 11:**
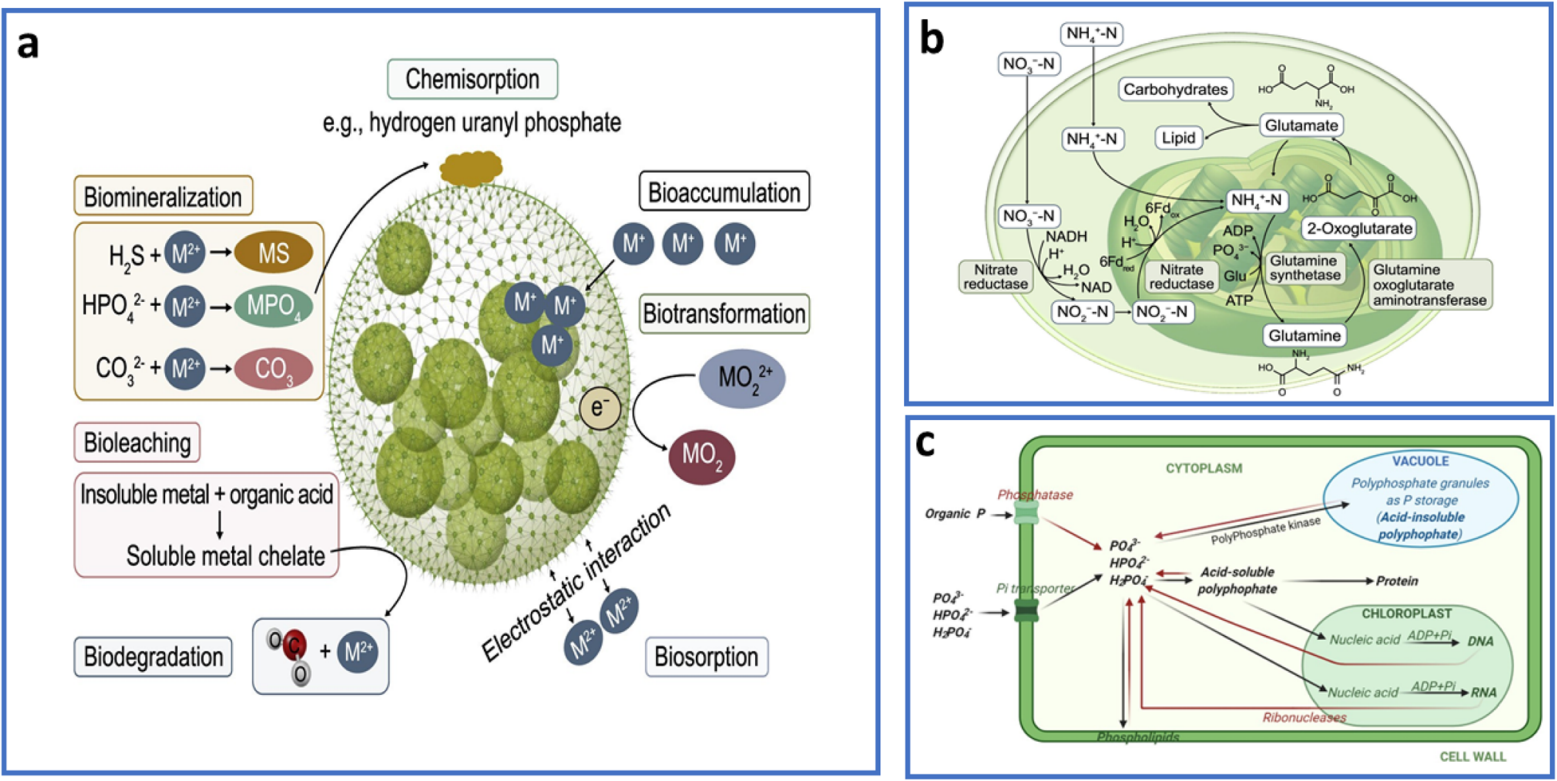
Metal–microbe interactions in bioremediation process (a) reproduced from ref. 185 with permission from Elsevier, *Environmental Science and Ecotechnology*, vol. **13**, p. 100205, Copyright 2023, mechanisms of nitrogen removal by microalgal cells in wastewater (b) reproduced from ref. 185 with permission from Elsevier, *Environmental Science and Ecotechnology*, vol. **13**, p. 100205, Copyright 2023, and schematic of phosphorus absorption and transformation pathway by microalgae (solid lines, under sufficient phosphorus conditions; dotted lines, under phosphorus deficiency conditions) (c) reproduced from ref. 235 with permission from Elsevier, *Science of the total Environment*, vol. **762**, p. 144590, Copyright 2021.

The use of algal consortia and the symbiotic relationship between bacteria and algae relative has been demonstrated to be more advantageous compared to using pure algal strain.^233^ Additionally, the combination of *Chlorella vulgaris* and biosurfactants has proven to be a superior approach for nutrient removal especially from petrochemical wastewaters.^234^ In activated sludge systems, bacteria decompose organic matter and yield CO_2_, which is consumed by algae during photosynthesis, expressing an excellent symbiotic relationship. During photosynthesis, microalgae generate oxygen, which serves as a crucial electron acceptor for aerobic bacterial degradation of pollutants. The exact phycoremediation pathways for nutrient removal may vary depending on the microalgal strains and consortia used, and include assimilation, biodegradation, sorption, and bioaccumulation, as shown in Fig. 11(b and c).

Recently, the performances of algae-activated sludge membrane bioreactor (AAS-MBR) and electro algae-activated sludge membrane bioreactor (e-AAS-MBR) has been compared with conventional MBR and e-MBR systems.^68^ The co-culture of algae and activated sludge increased NH_3_–N removal efficiencies of AAS-MBR and e-AAS-MBR 43.89 and 26.6% higher than that in the conventional MBR and e-MBR, respectively. Similarly, PO_4_^3−^–P removal efficiency was also found to be 6.43 and 2.66% higher in AAS-MBR and e-AAS-MBR relative to their counterparts. A significant reduction in membrane biofouling (57.30–61.95%) was also observed in both systems. Further modification in AMBR systems were achieved the performance evaluation of a microalgal-activated sludge membrane bioreactor (MAS-MBR) as a self-biological treatment or as a post-treatment for conventional biological treatments. Remarkably, high removal efficiencies of 94.36 ± 3.5% for ammonium and 88.37 ± 3% for phosphorus were achieved.^93^ Additionally, a lab scale AMBR, operating under 12 h dark/light cycle in continuous experiments, was investigated for nutrients removal and the reduction of anionic surfactants and in biofouling.^226^ The algal assimilation achieved a total nitrogen removal of 52% and total phosphorus removal of 36% however, the presence of nitrite (NO_3_–N) contents (>85%) in the effluent depicted that the nitrification and denitrification processes did not occur in the AMBR. Bacterial oxidation resulted in a 96% removal of BOD and 99% removal of anionic surfactants without requiring any external aeration source. The same group of researchers evaluated the effect of organic loading rate on the performance of microalgal MBRs to treat synthetic wastewater.^236^ Microalgal MBRs achieved up to 94% organic removal through bacterial oxidation without external aeration. Total nitrogen (TN) and total phosphorus (TP) removal rates with increasing organic loading rate (OLR). The highest TN (68.4%) and TP removal (37.7%) were achieved at an OLR of 0.014 kg dm^−3^. Further enhancement in nutrient removal could be accomplished through the deployment of hybrid bioreactors. For instance, a group of researchers has explored the performance in terms of oxygen production and nutrient utilization of an algal strain *Chlorella vulgaris* at different organic/inorganic carbon (OC/IC) and ammonium/nitrate (NH_4_^+^–N/NO^3−^–N) ratios in a hybrid aerobic membrane bioreactor (MBR) and membrane photobioreactor (MPBR) system.^228^ The findings revealed that 100% removal of PO_4_^3−^–P, 75% and 27% removal pf NH_4_^+^–N, and NO_3_–N, respectively was achieved. The performance evaluation of different AMBR systems in terms of nutrients removal is summarized in Table 2.

### 5.6. Miscellaneous applications of AMBRs

Beyond wastewater treatment, AMBRs hold significant potential for producing various value-added products, including biofuels such as biodiesel, bioethanol, and biogas, as well as high-valued compounds (*i.e.*, pigments, antioxidants, and pharmaceuticals). Additionally, AMBRs contribute to the production of animal feed and nutrient-rich fertilizer.^70,237,238^ Algal biomass serves as a promising feedstock for biofuel production, though challenges remain in achieving cost competitiveness with conventional fuels.^239^

AMBRs also play a pivotal role in carbon capture by utilizing carbon dioxide from flue gases and industrial processes, thereby reducing greenhouse gas emissions. In aquaculture, they support the farming of algae and aquatic plants, providing a sustainable food source for fish and other organisms residing in water. Furthermore, AMBRs effluents serve as a nutrient-rich fertilizers for agriculture and horticulture applications.^240^

**Fig. 12:**
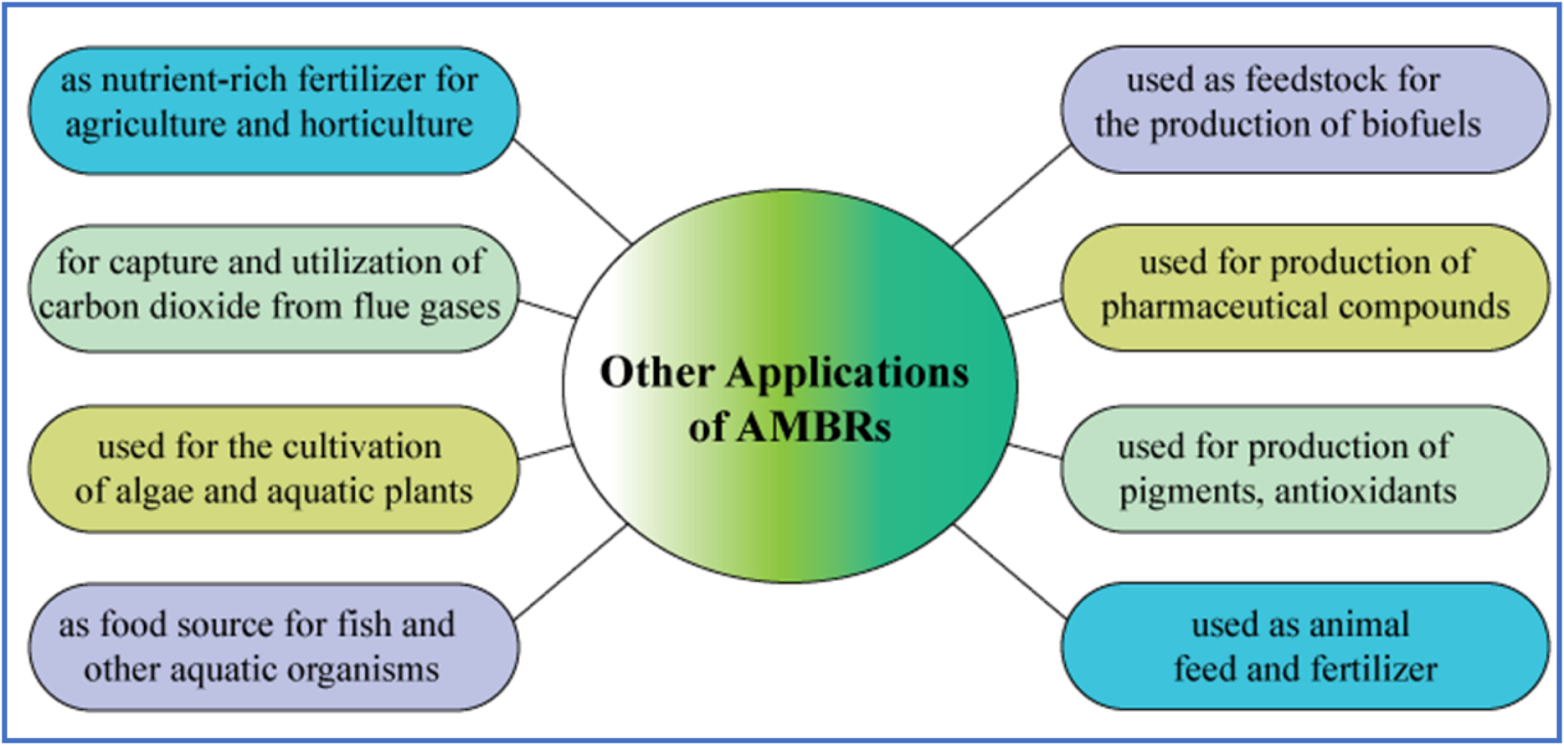
Miscellaneous applications of AMBRs.

A number of variables, including product value, market demand, production efficiency, and operating expenses, affect these applications' feasibility and economic potential. While some value-added products derived from AMBRs have gained commercial success, large-scale biofuel production remains economically challenging.^241,242^ However, ongoing technological advancements, optimized processes, and market development continue the applicability of AMBR for their extensive implementation and commercialization. A brief illustration of diverse applications of AMBRs is given in Fig. 12.

The selection of biomass valorization routes strongly depends on the wastewater composition and reactor configuration. Municipal or nutrient-rich effluents typically yield protein-rich biomass suitable for biofertilizers or animal feed, whereas high-carbon industrial effluents favor lipid accumulation for biofuel production.^243^ Systems treating pharmaceutical or metal-bearing wastewaters often produce biomass enriched with specific metabolites or bound metals, guiding its use toward bioproduct recovery rather than feed applications. Fig. 12 illustrates these relationships between feed characteristics, AMBR configuration, and downstream utilization.

## 6. Economic viability of AMBRs

Algal-based membrane bioreactors provide an ecologically sound substitute to traditional wastewater treatment processes for the elimination of ECs, although their economic feasibility is still being investigated. In contrast to conventional MBRs, AMBRs have the ability to lower operating expenses by eliminating the requirement for mechanical air circulation, as microalgae create oxygen spontaneously during photosynthesis. This can result in energy reductions of up to 30–50%, which is substantially high, considering that aeration accounts for a large portion of the total consumption of energy in MBRs. In addition, AMBRs help with recuperation of nutrients and carbon dioxide collection, giving ecological and financial benefits. However, the initial investment of AMBRs is often greater due to the requirement for dedicated photobioreactor infrastructures illumination systems, and the difficulties associate with preserving ideal algal conditions for their optimized development. Membrane fouling, which is aggravated by algal biomass buildup, increases up maintenance costs. Regardless of these hurdles, the financial picture increases when collected algal biomass is considered, since it may be transformed into biofuels, livestock feed, or valuable bioproducts like pigments and medicines. According to life cycle cost evaluations, while present AMBR systems are not currently financially-competitive at large-scale, technical developments, new membrane components, and method coupling with current wastewater systems might drastically reduce expenses over time. Reported capital costs for AMBRs typically range from USD 1.2–2.0 million per minimal liquid discharge (MLD), which is significantly lower than the 1.8–2.5 million per MLD reported for conventional MBRs.^244^ Low operating costs can be achieved by addressing reduced aeration and sludge handling requirements.^245^ Energy consumption generally lies between 0.45 and 0.8 kWh m^−3^, compared with 0.8–1.1 kWh m^−3^ for standard MBRs. These attributes suggest that AMBRs can achieve comparable performance with modest energy and cost savings. In short, AMBRs are a promising option for ecologically sound wastewater management in the future due to their multifunctional advantages and capacity for recuperation of resources, even though they currently face financial limitations when compared with existing technologies.^246^

## 7. Future prospects and challenges

AMBRs have shown promise in eliminating toxic and hazardous pollutants from wastewater. Compared to traditional treatment methods, AMBRs offer several advantages, including low energy requirements, high removal efficiency, and the potential for value-added byproducts. Although AMBRs provide novel solutions, their dependence on algal species may be challenged due to possible fluctuation in algal development and effectiveness under different environmental circumstances, which might limit their general acceptance in wastewater treatment techniques. Several challenges must be addressed to fully realize their potential. Key obstacles may include biomass or inhibition of bacterial and algal cell. Overcoming these challenges requires research focused on improving reactor design, optimizing process parameters, integrating complementary technologies, and expanding applications beyond wastewater treatment.

Future advancements are likely to emphasize the development of advanced AMBRs combined with bioenergy production. Research should also explore novel materials, including improved membranes and microbial consortia, as well as hybrid treatment methods. Despite progress in incorporating phosphate-solubilizing bacteria (PSB)and microalgae into MBRs for wastewater treatment, the metabolic activity of common bacteria remains relatively low in practical applications. Thus, further efforts are needed to filter and cultivate efficient microbial strains for treating refractory industrial effluent. The future prospects for AMBRs in sewage treatment are encouraging, especially in terms of tackling intriguing contaminants. However, continuous research is required to develop algal–bacterial combinations and increase operational specifications including HRT and material loading rates in attempt to improve pollution removal capacity. Incorporating sophisticated treatment technologies, such as forward osmosis and nanotechnology, might also improve the efficiency and long-term viability of AMBRs. Life cycle studies and techno-economic analyses will be critical in establishing the feasibility of AMBRs as opposed to traditional techniques, confirming that they are both financially and ecologically viable.

Over the past two decades, significant progress in genetic engineering has enabled the development of highly efficient microbial strains. These developments will facilitate more effective and streamlined solutions to existing challenges. However, maintaining stable and efficient treatment in MBR systems under extreme environmental conditions, such as a wide pH range and high salinity loading, remains a significant challenge. Additionally, membrane biofouling in high biomass environments significantly limits the widespread application of MBR technology.

Microorganism immobilization technologies offer a promising approach to mitigating these problems. Efforts should also be directed on lowering maintenance and operating costs and enhancing commercial viability and scalability. Despite these difficulties, AMBRs have extremely bright futures in the wastewater treatment industry. Innovative technologies like AMBRs are crucial for tackling these issues as water scarcity and pollution become more urgent worldwide concerns. To enable their broad adoption and optimize their impact in wastewater treatment, AMBRs require ongoing support and funding for research and development. Furthermore, solving difficulties such as membrane fouling and harvesting performance is critical for developing AMBR systems for commercial use. In conclusion, AMBRs provide a practical and sustainable solution to the growing demands of water resource management and wastewater treatment. Ultimately, the emergence of AMBRs has the potential to significantly contribute to the sustainable economy and recuperation of resources in the handling of wastewater.

## 8. Conclusions

This research provides an in-depth analysis of the increasing integration of microalgae in membrane bioreactors (MBR) for the removal of industrial wastewater and other contaminants. AMBRs have demonstrated efficient removal of EPs, such as personal care products and pathogens, even at shorter hydraulic retention times compared to conventional municipal wastewater treatment facilities. This suggests that AMBRs could be commercially implemented while requiring less space.

Since municipal wastewater treatment plants typically produce effluents with low BOD, COD, and TSS, AMBRs offer a viable solution for handling EPs. Additionally, AMBRs help prevent antibiotic-resistance bacteria from contaminating microalgal cultures while preserving biomass within the hybrid system. These reactors can potentially produce 50–100 mg per liter of algae per day, with phosphorus and nitrogen removal efficiencies ranging from 23–98% and 21–97%, respectively. Looking forward, algal-based membranes and AMBR systems hold strong potential for sustainable wastewater treatment and nutrient recovery. Their biological and physical synergy enables efficient removal of nutrients, organics, and emerging pollutants at lower energy costs. Key benefits include reduced sludge generation, self-supplied oxygen through algal photosynthesis, and opportunities for biomass valorization. However, some serious drawbacks (*i.e.*, membrane fouling from extracellular biopolymers, limited durability of polymeric membranes, uneven light distribution, and scale-up challenges) associated with AMBR technology need to be significantly addressed. Future research progress may rely on anti-fouling surface modifications, photoactive and hybrid ceramic polymeric membranes, and improved reactor hydrodynamics to enable stable and large-scale applications in pollutants removal.

## Author contributions

Nadeem Raza: supervision, writing original draft, reviewing, and editing, funding acquisition. Zeeshan Ali: project administration, writing – review & editing, supervision. Khalid Aziz: writing original draft, reviewing, and editing, Suryyia Manzoor, Anis Ahmad Chaudhary: reviewing and editing, data curation, investigation, Abdelmonaim Azzouz: formal analysis, and data curation. Surfaraz Hashim: reviewing and editing. Mostafa E. Salem & M. Khairy: data curation, reviewing and editing.

## Conflicts of interest

The authors declare that they have no known competing financial interests or personal relationships that could have appeared to influence the work reported in this paper.

## Data Availability

No new data were created or analyzed in this study. Data sharing is not applicable to this article as it is a review of previously published literature.

## References

[cit1] Aziz K., Raza N., Kanwal N., Khairy M., Ahmadi Y., Kim K.-H. (2025). Mater. Horiz..

[cit2] Qadir M. , Drechsel P., Jiménez Cisneros B., Kim Y., Pramanik A., Mehta P. and Olaniyan O., 2020, 44, pp. 40–51

[cit3] Lodh A., Shafi M., Goel S. (2025). Environ. Pollut..

[cit4] Aziz K., Naz A., Raza N., Manzoor S., Kim K.-H. (2024). Environ. Res..

[cit5] Sebastià P., Hasan S. W., Naddeo V. (2022). Case Stud. Chem. Environ. Eng..

[cit6] Ali Y. A. E. H., Azzouz A., Ahrouch M., Lamaoui A., Raza N., Lahcen A. A. (2024). J. Environ. Chem. Eng..

[cit7] Zahmatkesh S., Karimian M., Pourhanasa R., Ghodrati I., Hajiaghaei-Keshteli M., Ismail M. A. (2023). Chemosphere.

[cit8] Wijaya J., Oh S. (2023). Environ. Res..

[cit9] Al-Asheh S., Bagheri M., Aidan A. (2021). Case Stud. Chem. Environ. Eng..

[cit10] Paul A. , Dasgupta D., Hazra S., Chakraborty A., Haghighi M. and Chakraborty N., in Membranes for Water Treatment and Remediation, Springer, 2023, pp. 133–155

[cit11] Asante-Sackey D., Rathilal S., Tetteh E. K., Armah E. K. (2022). Membranes.

[cit12] Mohapatra M. , Mishra A. and Ray L., Biotechnological Removal of Emerging Pollutants from Wastewater Systems, 2025, vol. 245

[cit13] Sathya K., Nagarajan K., Carlin Geor Malar G., Rajalakshmi S., Raja Lakshmi P. (2022). Appl. Water Sci..

[cit14] Chandrappa R. and Das D. B., in Solid Waste Management: Principles and Practice, Springer, 2024, pp. 369–420

[cit15] Samanta N. S. , in Recent Trends in Industrial Wastewater Treatment, CRC Press, 2025, pp. 1–16

[cit16] Gondi R., Kavitha S., Kannah R. Y., Karthikeyan O. P., Kumar G., Tyagi V. K., Banu J. R. (2022). Bioresour. Technol..

[cit17] Long S., Hamilton P. B., Wang C., Li C., Xue X., Zhao Z., Wu P., Gu E., Uddin M. M., Li B. (2024). J. Hazard. Mater..

[cit18] Kumar A., Nighojkar A., Varma P., Prakash N. J., Kandasubramanian B., Zimmermann K., Dixit F. (2023). J. Hazard. Mater..

[cit19] Ethiraj S., Samuel M. S., Indumathi S. (2024). Biocatal. Agric. Biotechnol..

[cit20] Ratnasari A., Syafiuddin A., Zaidi N. S., Kueh A. B. H., Hadibarata T., Prastyo D. D., Ravikumar R., Sathishkumar P. (2022). Environ. Pollut..

[cit21] Satpati G. G., Kundu D., Rajak R. C., Gupta S., Kim J.-W., Davoodbasha M. (2024). Algal Res..

[cit22] AlHani H. A. , Physiological adaptation of unicellular microalgae to environmental stress and their potential for biofuel production, Doctoral dissertation, University of Sheffield, 2019.

[cit23] Leong Y. K., Huang C.-Y., Chang J.-S. (2021). J. Environ. Manage..

[cit24] Abreu A. P., Morais R. C., Teixeira J. A., Nunes J. (2022). Renew. Sustain. Energy Rev..

[cit25] Hejna M., Kapuścińska D., Aksmann A. (2022). Int. Res. J. Publ. Environ. Health.

[cit26] Shanmuganathan R., Kadri M. S., Mathimani T., Le Q. H., Pugazhendhi A. (2023). Chemosphere.

[cit27] Liu X.-y., Hong Y. (2021). Curr. Pollut. Rep..

[cit28] Leng L., Wei L., Xiong Q., Xu S., Li W., Lv S., Lu Q., Wan L., Wen Z., Zhou W. (2020). Chemosphere.

[cit29] Liu X., Sathishkumar K., Zhang H., Saxena K. K., Zhang F., Naraginiti S., Rajendiran R., Aruliah R., Guo X. (2024). J. Hazard. Mater. Adv..

[cit30] Javed M. U., Mukhtar H., Zieniuk B., Rashid U. (2024). Fermentation.

[cit31] Nath S., Astapati A. D., Naha A., Sharma I., Shah M. P. (2024). Curr. Res. Biotechnol..

[cit32] Yuliasni R. , Kurniawan S. B., Marlena B., Hidayat M. R., Kadier A. and Ma P. C., Phycoremediation Processes in Industrial Wastewater Treatment, 2023, pp. 21–41

[cit33] Wibowo Y. G., Syahnur M. T., Al-Azizah P. S., Gintha D. A., Lululangi B. R. G. (2023). Environ. Nanotechnol. Monit. Manag..

[cit34] Sugumaran R., Padam B. S., Yong W. T. L., Saallah S., Ahmed K., Yusof N. A. (2022). Int. Res. J. Publ. Environ. Health.

[cit35] Fernand F., Israel A., Skjermo J., Wichard T., Timmermans K. R., Golberg A. (2017). Renew. Sustain. Energy Rev..

[cit36] Jha S., Mishra B. K. (2024). Environ. Sci. Pollut. Res..

[cit37] Kishore S., Malik S., Shah M. P., Bora J., Chaudhary V., Kumar L., Sayyed R. Z., Ranjan A. (2024). Biotechnol. Genet. Eng. Rev..

[cit38] Dewali S. , Parveen N., Kathayat N., Rawat G., Bora S., Sharma N. P., Pathak V. M. and Bisht S. S., in Emerging Contaminants in Water and Wastewater: Sources and Substances, Springer, 2025, pp. 255–279

[cit39] Alwin A., Cahyono T., Sya'ban A., Dahlia S. (2023). J. Environ. Sci..

[cit40] He Z., Li Y., Qi B. (2022). Environ. Sci. Pollut. Res..

[cit41] Gurau S., Imran M., Ray R. L. (2024). Environ. Technol. Innovat..

[cit42] Wang X., Li S.-Y., Chen Y.-P., Guo J.-S., Liu S.-Y., Yan P. (2024). Chem. Eng. J..

[cit43] Hussain F., Shah S. Z., Ahmad H., Abubshait S. A., Abubshait H. A., Laref A., Manikandan A., Kusuma H. S., Iqbal M. (2021). Renew. Sustain. Energy Rev..

[cit44] Mofijur M., Hasan M., Sultana S., Kabir Z., Djavanroodi F., Ahmed S. F., Jahirul M., Badruddin I. A., Khan T. Y. (2023). Chemosphere.

[cit45] Bera S. P., Godhaniya M., Kothari C. (2022). J. Basic Microbiol..

[cit46] Chatterjee B., Baruah S., Chatterjee D., Dey S., Mitra A. K. (2024). Clean: Soil, Air, Water.

[cit47] Saini N., Dhull P., Pal M., Manzoor I., Rao R., Mushtaq B., Aamir M. (2024). J. Environ. Chem. Eng..

[cit48] Kofler J. R., Labeeuw L., Bates H., Zavafer A., Ralph P. J. (2023). Algal Res..

[cit49] Wang X., Zhou Y., Peng Q., Han Y., Yang J., Xu H., Li C., Li L., Dou S., Yang M. (2022). Algal Res..

[cit50] Zhou H., Xu Z., Zhou L., Zhang Z., Wang J., Lan C. Q. (2023). Biochem. Eng. J..

[cit51] Siddiqui S. A., Wu Y. S., Saikia T., Ucak İ., Afreen M., Shah M. A., Ayivi R. D. (2023). Environ. Chem. Lett..

[cit52] Ghazvini M., Kavosi M., Sharma R., Kim M. (2022). Biomass Bioenergy.

[cit53] Delanka-Pedige H. M. K., Munasinghe-Arachchige S. P., Abeysiriwardana-Arachchige I. S. A., Nirmalakhandan N. (2021). J. Clean. Prod..

[cit54] Abudaqqa W. S., Madhuranthakam C. M. R., Chaalal O. (2024). J. Water Proc. Eng..

[cit55] Kotobuki M., Gu Q., Zhang L., Wang J. (2021). Molecules.

[cit56] Lesimple A., Jasim S. Y., Johnson D. J., Hilal N. (2020). J. Water Proc. Eng..

[cit57] Eloffy M., El-Sherif D. M., Abouzid M., Elkodous M. A., El-nakhas H. S., Sadek R. F., Ghorab M. A., Al-Anazi A., El-Sayyad G. S. (2021). Nanotechnol. Rev..

[cit58] Pulido-Aponte A. , Sangregorio-Soto V., and Garzón-Castro C. L., Monitoring and Control System for Closed Microalgae Cultures at Pilot Scale, in 13th International Symposium on Advanced Topics in Electrical Engineering (ATEE), IEEE, 2023, pp. 1–6.

[cit59] Nchindia F. E. (2022). IET Renew. Power Gener..

[cit60] Kong W., Kong J., Feng S., Yang T., Xu L., Shen B., Bi Y., Lyu H. (2024). Biotechnol. Biofuels Bioprod..

[cit61] Vo T. P., Danaee S., Chaiwong C., Pham B. T., Kim M., Kuzhiumparambil U., Songsomboon C., Pernice M., Ngo H. H., Ralph P. J. (2024). J. Environ. Chem. Eng..

[cit62] Saravanan A., Kumar P. S., Varjani S., Jeevanantham S., Yaashikaa P., Thamarai P., Abirami B., George C. S. (2021). Chemosphere.

[cit63] Oruganti R. K., Katam K., Show P. L., Gadhamshetty V., Upadhyayula V. K. K., Bhattacharyya D. (2022). Bioengineered.

[cit64] Galang M. G. K., Chen J., Cobb K., Zarra T., Ruan R. (2025). Environ. Technol..

[cit65] Tang C.-C., Hu Y.-R., He Z.-W., Li Z.-H., Tian Y., Wang X. C. (2024). Chem. Eng. J..

[cit66] Jin D., Zhang X., Zhou L., Zhang X., Wu P. (2024). J. Water Proc. Eng..

[cit67] Wu P.-H., Hsieh T.-M., Wu H.-Y., Yu C.-P. (2021). Int. Biodeterior. Biodegrad..

[cit68] Corpuz M. V. A., Borea L., Senatore V., Castrogiovanni F., Buonerba A., Oliva G., Ballesteros Jr F., Zarra T., Belgiorno V., Choo K.-H. (2021). Sci. Total Environ..

[cit69] Kwakye J. M., Ekechukwu D. E., Ogbu A. D. (2024). Renewable Sustainable Energy Rev..

[cit70] Radmehr S., Peltomaa E., Kallioinen-Mänttäri M., Mänttäri M. (2023). Bioresour. Technol..

[cit71] Solmaz A., Işık M. (2020). Biomass Bioenergy.

[cit72] Manu L., Mokolensang J. F., Gunawan W. B., Setyawardani A., Salindeho N., Syahputra R. A., Iqhrammullah M., Nurkolis F. (2024). J. Agric. Food Res..

[cit73] Udayan A., Sirohi R., Sreekumar N., Sang B.-I., Sim S. J. (2022). Bioresour. Technol..

[cit74] Sreeharsha R. V. and Venkata Mohan S., in Microbial Photosynthesis: from Basic Biology to Artificial Cell Factories and Industrial Applications, Springer, 2024, pp. 57–80

[cit75] Wu W., Tan L., Chang H., Zhang C., Tan X., Liao Q., Zhong N., Zhang X., Zhang Y., Ho S.-H. (2023). Renew. Sustain. Energy Rev..

[cit76] Gupta S. K. , Dhandayuthapani K. and Ansari F. A., Phytomanagement of Polluted Sites, 2019, pp. 471–499

[cit77] Elsayad R. M., Sharshir S. W., Khalil A., Basha A. M. (2024). J. Environ. Manage..

[cit78] Safdar W. , Qazi A. S., Ahmed S., Tariq M. R. and Ahmed H., in Pharmaceutical and Nutraceutical Potential of Cyanobacteria, Springer, 2024, pp. 161–194

[cit79] Zou H., Rutta N. C., Chen S., Zhang M., Lin H., Liao B. (2022). Membranes.

[cit80] García-Galán M. J., Gutiérrez R., Uggetti E., Matamoros V., García J., Ferrer I. (2018). Biosyst. Eng..

[cit81] Marbelia L., Bilad M. R., Passaris I., Discart V., Vandamme D., Beuckels A., Muylaert K., Vankelecom I. F. (2014). Bioresour. Technol..

[cit82] Torzillo G. and Chini Zittelli G., Algal Biorefineries: Volume 2: Products and Refinery Design, 2015, pp. 187–212

[cit83] Tekere M., Jacob-Lopes E., Zepka L. Q. (2019). Biotechnol. Bioeng..

[cit84] Mojiri A., Baharlooeian M., Kazeroon R. A., Farraji H., Lou Z. (2020). Microorganisms.

[cit85] Verma R., Kumar R., Mehan L., Srivastava A. (2018). J. Biosci. Bioeng..

[cit86] Mohan S. V. , Devi M. P., Subhash G. V. and Chandra R., in Biofuels from Algae, Elsevier, 2014, pp. 155–187

[cit87] Ismail M. M., Essam T. M., Ragab Y. M., El-Sayed A. E.-K. B., Mourad F. E. (2017). Bioresour. Technol..

[cit88] Wang X. and Hong Y., in Algal Bioreactors, Elsevier, 2025, pp. 685–694

[cit89] Dey S., Samanta P., Ghosh A. R., Banerjee S., Sen K. (2024). Clean. Water.

[cit90] Mujtaba G., Rizwan M., Lee K. (2015). Biotechnol. Bioproc. Eng..

[cit91] Movahed E., Saeb K., Pajoum Shariati F., Rahnavard A. (2022). J. Environ. Eng..

[cit92] Takdastan A., Talepour N., Taherian M. (2025). Environ. Technol. Rev..

[cit93] Chaleshtori S. N., Shamskilani M., Babaei A., Behrang M. (2022). J. Water Proc. Eng..

[cit94] Xiao X., Guo H., Ma F., You S., Geng M., Kong X. (2021). Sci. Total Environ..

[cit95] Aslam Z., Alam P., Islam R., Khan A. H., Samaraweera H., Hussain A., Zargar T. I. (2024). J. Taiwan Inst. Chem. Eng..

[cit96] Jijingi H. E., Yazdia S. K., Abakr Y. A., Etim E. (2024). Case Stud. Chem. Environ. Eng..

[cit97] Uddin M., Islam M. K., Dev S. (2024). Heliyon.

[cit98] Saidulu D., Majumder A., Gupta A. K. (2021). J. Environ. Chem. Eng..

[cit99] Tecirli E. S., Akgun K., Caglak A., Sari Erkan H., Onkal Engin G. (2024). Water Environ. J..

[cit100] Eshamuddin M., Zuccaro G., Nourrit G., Albasi C. (2024). J. Environ. Chem. Eng..

[cit101] Di Bella G., Mannina G. (2020). Water.

[cit102] Tian J.-Y., Liang H., Nan J., Yang Y.-L., You S.-J., Li G.-B. (2009). Chem. Eng. J..

[cit103] Low S. L., Ong S. L., Ng H. Y. (2016). Chem. Eng. J..

[cit104] Singh R. , Membrane Technology and Engineering for Water Purification: Application, Systems Design and Operation, Butterworth-Heinemann, 2014

[cit105] Najmi M., Mehrnia M. R., Tashauoei H. R., Iranpoury A., Alivand M. S. (2020). J. Environ. Chem. Eng..

[cit106] Mustafa Z. Z., Murdock A. T., Xie Z., Johnston-Hall G., Henderson R. K., Leslie G. L., Le-Clech P. (2025). Sep. Purif. Technol..

[cit107] Wan N., Shi J., Zhou P., Zhang X., Zhang X., Huang Y., Liu J. (2023). J. Water Proc. Eng..

[cit108] Heo J., Kwon D., Beirns E., Tan G.-Y. A., Lee P.-H., Kim J. (2023). J. Environ. Chem. Eng..

[cit109] Xiao J., Qaisar M., Zhu X., Li W., Zhang K., Liang N., Feng H., Cai J. (2025). J. Environ. Manage..

[cit110] Menon S. S., Kalyanraman V. (2025). Environ. Technol. Rev..

[cit111] Sandoval-García V., Ruano M., Alliet M., Brepols C., Comas J., Harmand J., Heran M., Mannina G., Rodriguez-Roda I., Smets I. (2025). Water Res..

[cit112] Ramesh B., Saravanan A., Kumar P. S., Yaashikaa P., Thamarai P., Shaji A., Rangasamy G. (2023). Environ. Pollut..

[cit113] Rani S., Gunjyal N., Ojha C., Singh R. P. (2021). J. Hazard. Toxic Radioact. Waste.

[cit114] Shafiquzzaman M., Haider H., Ashadullah A. (2021). Process Saf. Environ. Prot..

[cit115] Novoa A. F., Vrouwenvelder J. S., Fortunato L. (2021). Front. Chem. Eng..

[cit116] Zhang Z., Chen M., Li J., Zhao B., Wang L. (2020). Arab. J. Chem..

[cit117] Xu M., Wang X., Zhou B., Zhou L. (2021). J. Hazard. Mater..

[cit118] Zhang G., Ji S., Gao X., Liu Z. (2008). J. Membr. Sci..

[cit119] Sohn W., Guo W., Ngo H. H., Deng L., Cheng D., Zhang X. (2021). J. Water Proc. Eng..

[cit120] Nath A., Mishra A., Pande P. P. (2021). Mater. Today: Proc..

[cit121] Gu Y., Li Y., Li X., Luo P., Wang H., Robinson Z. P., Wang X., Wu J., Li F. (2017). Appl. Energy.

[cit122] Mohan S. M., Nagalakshmi S. (2020). J. Water Proc. Eng..

[cit123] Sun L., Tian Y., Li H., Wang Q. (2021). Environ. Int..

[cit124] Fu Y., Li S.-Y., Chen Y., Chen Y.-P., Guo J.-S., Liu S.-Y., Yan P. (2024). Bioresour. Technol..

[cit125] Zhao Z., Liu B., Ilyas A., Vanierschot M., Muylaert K., Vankelecom I. F. (2021). J. Membr. Sci..

[cit126] Lutzu G. A., Ciurli A., Chiellini C., Di Caprio F., Concas A., Dunford N. T. (2021). J. Environ. Chem. Eng..

[cit127] Nagarajan D., Lee D.-J., Varjani S., Lam S. S., Allakhverdiev S. I., Chang J.-S. (2022). Sci. Total Environ..

[cit128] Jiang L., Li Y., Pei H. (2021). Renew. Sustain. Energy Rev..

[cit129] Wang Y., He Y., Li X., Nagarajan D., Chang J.-S. (2022). Bioresour. Technol..

[cit130] Nascimento T. A., Fdz-Polanco F., Peña M. (2020). Separ. Purif. Rev..

[cit131] Dubey D. and Dutta V., Environmental Concerns and Sustainable Development: Volume 2: Biodiversity, Soil and Waste Management, 2020, pp. 81–126

[cit132] Holloway R. W., Achilli A., Cath T. Y. (2015). Environ. Sci.: Water Res. Technol..

[cit133] Sutherland K. (2010). Filtrat. Separ..

[cit134] Zahmatkesh S., Karimian M., Pourhanasa R., Ghodrati I., Hajiaghaei-Keshteli M., Ismail M. A. (2023). Chemosphere.

[cit135] Zhang X., Fan R., Xu Y., Gao Y.-Z., Bizimana A., Naidoo A. R., Han B.-C., Meng X.-Z. (2022). Separations.

[cit136] Sarma U., Hoque M. E., Thekkangil A., Venkatarayappa N., Rajagopal S. (2024). J. Hazard. Mater. Adv..

[cit137] El-Sheekh M. M., El-Kassas H. Y., Ali S. S. (2025). Microb. Cell Factories.

[cit138] Samal K., Mahapatra S., Ali M. H. (2022). Energy Nexus.

[cit139] Zhang R., Hao L., Cheng K., Xin B., Sun J., Guo J. (2023). Chemosphere.

[cit140] Dawen G. and Nabi M., in Novel Approaches towards Wastewater Treatment: Effective Strategies and Techniques, Springer, 2024, pp. 315–416

[cit141] Singh P. K., Kumar U., Kumar I., Dwivedi A., Singh P., Mishra S., Seth C. S., Sharma R. K. (2024). Environ. Sci. Pollut. Res..

[cit142] Sangion A., Gramatica P. (2016). Environ. Int..

[cit143] Guo W. , Cheng D., Ngo H. H., Chang S. W., Nguyen D. D., Nguyen D. P. and Bui X. T., in Current Developments in Biotechnology and Bioengineering, Elsevier, 2020, pp. 219–239

[cit144] Rajabi M., Keihankhadiv S., Suhas, Tyagi I., Karri R. R., Chaudhary M., Mubarak N. M., Chaudhary S., Kumar P., Singh P. (2023). J. Nanostruct. Chem..

[cit145] Wani A. K., ul Gani Mir T., Akhtar N., Chopra C., Bashir S. M., Hassan S., Kumar V., Singh R., Américo-Pinheiro J. H. P. (2024). Curr. Microbiol..

[cit146] Shamshad J., Rehman R. U. (2025). Environ. Sci. Adv..

[cit147] Zhang J., Yang H., Sun Y., Yan B., Chen W., Fan D. (2024). Compr. Rev. Food Sci. Food Saf..

[cit148] Ong H. C., Tiong Y. W., Goh B. H. H., Gan Y. Y., Mofijur M., Fattah I. R., Chong C. T., Alam M. A., Lee H. V., Silitonga A. S. (2021). Energy Convers. Manage..

[cit149] Singh S., Pant A., Dutta K., Rani R., Vithanage M., Daverey A. (2024). J. Environ. Chem. Ecotoxicol..

[cit150] Ren H., Wang R., Ying L., Iyobosa E., Chen G., Zang D., Tong M., Li E., Nerenberg R. (2025). Water Res..

[cit151] Mantovani M., Rossi S., Ficara E., Collina E., Marazzi F., Lasagni M., Mezzanotte V. (2024). Sci. Total Environ..

[cit152] Ndlela L. L., Schroeder P., Genthe B., Cruzeiro C. (2023). Toxics.

[cit153] Chu Y., Zhang C., Wang R., Chen X., Ren N., Ho S.-H. (2022). Water Res..

[cit154] Aydin S., Ünlü İ. D., Arabacı D. N., Duru Ö. A. (2022). Sci. Total Environ..

[cit155] Kiki C., Ye X., Li X., Adyari B., Hu A., Qin D., Yu C.-P., Sun Q. (2022). J. Hazard. Mater..

[cit156] Hom-Diaz A., Jaén-Gil A., Rodríguez-Mozaz S., Barceló D., Vicent T., Blánquez P. (2022). Algal Res..

[cit157] Rambaldo L., Ávila H., Casas M. E., Guivernau M., Viñas M., Trobajo R., Pérez-Burillo J., Mann D. G., Fernández B., Biel C. (2022). Chemosphere.

[cit158] Mojiri A., Zhou J. L., Nazari M., Rezania S., Farraji H., Vakili M. (2022). Process Saf. Environ. Prot..

[cit159] Hena S., Gutierrez L., Croué J.-P. (2020). J. Hazard Mater..

[cit160] Peng Y.-Y., Gao F., Yang H.-L., Li C., Lu M.-M., Yang Z.-Y. (2020). Sci. Total Environ..

[cit161] Xie P., Chen C., Zhang C., Su G., Ren N., Ho S.-H. (2020). Water Res..

[cit162] da Silva Rodrigues D. A., da Cunha C. C. R. F., Freitas M. G., de Barros A. L. C., e Castro P. B. N., Pereira A. R., de Queiroz
Silva S., da Fonseca Santiago A., Afonso R. J. D. C. F. (2020). Sci. Total Environ..

[cit163] Xiong Q., Liu Y.-S., Hu L.-X., Shi Z.-Q., Cai W.-W., He L.-Y., Ying G.-G. (2020). Water Res..

[cit164] Xiong J.-Q., Kurade M. B., Jeon B.-H. (2017). Chem. Eng. J..

[cit165] Kim M., Guerra P., Shah A., Parsa M., Alaee M., Smyth S. (2014). Water Sci. Technol..

[cit166] Peng F.-Q., Ying G.-G., Yang B., Liu S., Lai H.-J., Liu Y.-S., Chen Z.-F., Zhou G.-J. (2014). Chemosphere.

[cit167] Parladé E., Hom-Diaz A., Blánquez P., Martínez-Alonso M., Vicent T., Gaju N. (2018). Water Res..

[cit168] García-Galán M. J., Matamoros V., Uggetti E., Díez-Montero R., García J. (2021). Environ. Res..

[cit169] Escapa C., Coimbra R., Paniagua S., García A., Otero M. (2017). J. Environ. Manag..

[cit170] Matamoros V., Uggetti E., García J., Bayona J. M. (2016). J. Hazard Mater..

[cit171] Li Y., Yang X., Wong M., Geng B. (2023). Algal Res..

[cit172] Avila R., García-Vara M., López-García E., Postigo C., de Alda M. L., Vicent T., Blánquez P. (2022). Sci. Total Environ..

[cit173] Hu N., Xu Y., Sun C., Zhu L., Sun S., Zhao Y., Hu C. (2021). Ecotoxicol. Environ. Saf..

[cit174] Derakhshan Z., Ehrampoush M. H., Mahvi A. H., Dehghani M., Faramarzian M., Eslami H. (2019). Chem. Eng. J..

[cit175] Mollamohammada S., Aly Hassan A., Dahab M. (2021). Water Environ. Res..

[cit176] Quan L., Cheng Y., Wang J., Chen Y., Li D., Wang S., Li B., Zhang Z., Yang L., Wu L. (2023). J. Environ. Manage..

[cit177] Encarnação T., Santos D., Ferreira S., Valente A. J., Pereira J., Campos M., Burrows H. D., Pais A. A. (2021). Bull. Environ. Contam. Toxicol..

[cit178] García-Galán M. J., Monllor-Alcaraz L. S., Postigo C., Uggetti E., de Alda M. L., Díez-Montero R., García J. (2020). Environ. Pollut..

[cit179] Kurade M. B., Kim J. R., Govindwar S. P., Jeon B.-H. (2016). Algal Res..

[cit180] Sousa H., Sousa C. A., Vale F., Santos L., Simões M. (2023). Sci. Total Environ..

[cit181] Vale F., Sousa C. A., Sousa H., Santos L., Simões M. (2022). Chem. Eng. J..

[cit182] Mojiri A., Zhou J. L., Ratnaweera H., Rezania S., Nazari M. (2022). Chemosphere.

[cit183] Santaeufemia S., Abalde J., Torres E. (2019). J. Hazard Mater..

[cit184] Bai X., Acharya K. (2016). J. Hazard Mater..

[cit185] Abdelfattah A., Ali S. S., Ramadan H., El-Aswar E. I., Eltawab R., Ho S.-H., Elsamahy T., Li S., El-Sheekh M. M., Schagerl M. (2023). Environ. Sci. Biotechnol..

[cit186] Banjara R. A. , Kumar A., Aneshwari R. K. and Chandrawanshi N. K., in Microbes and Enzymes for Water Treatment and Remediation, CRC Press, 2024, pp. 13–44

[cit187] Priya A., Gnanasekaran L., Rajendran S., Qin J., Vasseghian Y. (2022). Environ. Res..

[cit188] Goh P. S., Lau W. J., Ismail A. F., Samawati Z., Liang Y. Y., Kanakaraju D. (2022). Water.

[cit189] Wang L., Xiao H., He N., Sun D., Duan S. (2019). Sci. Rep..

[cit190] Singh M. , Unadkat K., Parikh P. and Chandrasekhar K., in Pesticides Bioremediation, Springer, 2022, pp. 353–380

[cit191] Touliabah H. E.-S., El-Sheekh M. M., Ismail M. M., El-Kassas H. (2022). Molecules.

[cit192] Styszko K., Durak J., Malicka A., Bochnia T., Żaba T. (2021). Desalin. Water Treat..

[cit193] Alex R. K., Maes T., Devipriya S. P. (2024). Emerging Contam..

[cit194] Kamaz M., Wickramasinghe S. R., Eswaranandam S., Zhang W., Jones S. M., Watts M. J., Qian X. (2019). Int. Res. J. Publ. Environ. Health.

[cit195] Xu Y., Shui X., Gao M., Zhang Y., Zhang Z., Zhu Z., Zhao B., Sun D. (2024). J. Hazard. Mater..

[cit196] Tella T. A. , Festus B., Olaoluwa T. D. and Oladapo A. S., in Smart Nanomaterials for Environmental Applications, Elsevier, 2025, pp. 351–385

[cit197] Ali Z., Sajid M., Raza N., Sohail Y., Hayat M., Manzoor S., Shakeel N., Gill K. A., Ifseisi A. A., Ansari M. Z. (2023). Arab. J. Chem..

[cit198] Ghosh A., Sah D., Chakraborty M., Rai J. (2024). Carbohydr. Res..

[cit199] Sibiya N. P., Mahlangu T. P., Tetteh E. K., Rathilal S. (2024). Clean. Chem. Eng..

[cit200] Abinandan S., Subashchandrabose S. R., Panneerselvan L., Venkateswarlu K., Megharaj M. (2019). Bioresour. Technol..

[cit201] Rajalakshmi A., Silambarasan T., Dhandapani R. (2021). Appl. Water Sci..

[cit202] Saleem M., Alibardi L., Lavagnolo M. C., Cossu R., Spagni A. (2016). J. Environ. Manage..

[cit203] Fard G. H., Mehrnia M. R. (2017). J. Environ. Chem. Eng..

[cit204] Madhav S., Mishra R., Kumari A., Srivastav A., Ahamad A., Singh P., Ahmed S., Mishra P., Sillanpää M. (2024). Int. J. Environ. Sci. Technol..

[cit205] Naaz F., Bhattacharya A., Mathur M., Bano F., Pant K. K., Malik A. (2021). J. Water Proc. Eng..

[cit206] Sulaymon A. H., Mohammed A. A., Al-Musawi T. J. (2013). Desalination Water Treat..

[cit207] Mona S., Kaushik A. (2015). Ecol. Eng..

[cit208] Bhattacharya P., Jana S., Banerjee S. (2024). Water Sci. Technol..

[cit209] Jahandust M., Esmaeili A. (2024). RSC Adv..

[cit210] Guan C.-Y., Kao Y.-L., Wu P.-H., Yu C.-P. (2023). J. Environ. Chem. Eng..

[cit211] Mantovani M., Collina E., Lasagni M., Marazzi F., Mezzanotte V. (2023). Environ. Sci. Pollut. Res..

[cit212] Zhou H., Zhao X., Kumar K., Kunetz T., Zhang Y., Gross M., Wen Z. (2021). Algal Res..

[cit213] Yigit E., Yurtsever A., Basaran S. T., Sahinkaya E. (2020). Environ. Technol. Innovat..

[cit214] Shen Y., Zhu W., Li H., Ho S.-H., Chen J., Xie Y., Shi X. (2018). Bioresour. Technol..

[cit215] Ma L., Wang F., Yu Y., Liu J., Wu Y. (2018). Bioresour. Technol..

[cit216] Sulaymon A. H., Mohammed A. A., Al-Musawi T. J. (2013). Environ. Sci. Pollut. Res..

[cit217] Asaad A. A., Amer A. S. (2024). Sci. Rep..

[cit218] Ruan L., Xu D., Cheng M., Liang Y., Wu L., Zhang X., Zhang T., Huang Y., Guo C., Shang C. (2024). Waste Biomass Valoriz..

[cit219] Perumalsamy M. (2024). Biomass Convers. Biorefinery.

[cit220] Yan H., Chen Z., Ngo H. H., Wang Q.-P., Hu H.-Y. (2024). Bioresour. Technol..

[cit221] Fan J., Yuan W., Zhang X., Ji B., Du X. (2024). Sci. Total Environ..

[cit222] Sun P., Ji B., Li A., Zhang X., Liu Y. (2024). Bioresour. Technol..

[cit223] Salgado E. M., Esteves A. F., Gonçalves A. L., Pires J. C. (2023). Environ. Res..

[cit224] Theepharaksapan S., Lerkmahalikit Y., Namyuang C., Ittisupornrat S. (2023). J. Environ. Chem. Eng..

[cit225] Silva A. D., Fernandes D. F., Figueiredo S. A., Freitas O. M., Delerue-Matos C. (2022). Int. Res. J. Publ. Environ. Health.

[cit226] Shafiquzzaman M., Ashadullah A., Haider H., Hasan M. M., Azam M., Alresheedi M., AlSaleem S., Ghumman A. (2022). Int. J. Environ. Sci. Technol..

[cit227] Morillas-España A., Sánchez-Zurano A., Lafarga T., del Mar Morales-Amaral M., Gómez-Serrano C., Acién-Fernández F. G., González-López C. V. (2021). Algal Res..

[cit228] Najm Y., Jeong S., Leiknes T. (2017). Bioresour. Technol..

[cit229] Lee C. S., Lee S.-A., Ko S.-R., Oh H.-M., Ahn C.-Y. (2015). Water Res..

[cit230] Ma X., Zhou W., Fu Z., Cheng Y., Min M., Liu Y., Zhang Y., Chen P., Ruan R. (2014). Bioresour. Technol..

[cit231] Choix F. J., Bashan Y., Mendoza A., De-Bashan L. E. (2014). J. Biotechnol..

[cit232] Liang Z., Liu Y., Ge F., Xu Y., Tao N., Peng F., Wong M. (2013). Chemosphere.

[cit233] Chen X., Hu Z., Qi Y., Song C., Chen G. (2019). Bioresour. Technol..

[cit234] Madadi R., Pourbabaee A., Tabatabaei M., Zahed M., Naghavi M. (2016). Int. J. Environ. Res..

[cit235] Su Y. (2021). Sci. Total Environ..

[cit236] Ashadullah A., Shafiquzzaman M., Haider H., Alresheedi M., Azam M. S., Ghumman A. R. (2021). J. Environ. Manage..

[cit237] Baral S. S., Dionisi D., Maarisetty D., Gandhi A., Kothari A., Gupta G., Jain P. (2020). Biomass Bioenergy.

[cit238] Ahmad A. , Banat F. and Taher H., in Algal Biotechnology, Elsevier, 2022, pp. 3–32

[cit239] Kumar M., Sun Y., Rathour R., Pandey A., Thakur I. S., Tsang D. C. (2020). Sci. Total Environ..

[cit240] Sharma N. K. and Arivalagan A. R., in Handbook of Advanced Approaches towards Pollution Prevention and Control, Elsevier, 2021, pp. 217–247

[cit241] Siddiki S. Y. A., Mofijur M., Kumar P. S., Ahmed S. F., Inayat A., Kusumo F., Badruddin I. A., Khan T. Y., Nghiem L., Ong H. C. (2022). Fuel.

[cit242] Bhatt P., Bhandari G., Turco R. F., Aminikhoei Z., Bhatt K., Simsek H. (2022). Environ. Pollut..

[cit243] Rai S. K., Kim G., Song H. (2025). Catalysts.

[cit244] Raja W., Kumar P. (2025). Indian Chem. Eng..

[cit245] Omokaro G. O., Nafula Z. S., Iloabuchi N. E., Chikukula A. A., Osayogie O. G., Nnoli E. C. (2025). Sustain. Chem. Clim. Action.

[cit246] Malik S., Dhasmana A., Preetam S., Mishra Y. K., Chaudhary V., Bera S. P., Ranjan A., Bora J., Kaushik A., Minkina T. (2022). Nanomaterials.

